# Mifepristone Increases Life Span of Virgin Female *Drosophila* on Regular and High-fat Diet Without Reducing Food Intake

**DOI:** 10.3389/fgene.2021.751647

**Published:** 2021-09-24

**Authors:** Gary N. Landis, Tyler A. U. Hilsabeck, Hans S. Bell, Tal Ronnen-Oron, Lu Wang, Devon V. Doherty, Felicia I. Tejawinata, Katherine Erickson, William Vu, Daniel E. L. Promislow, Pankaj Kapahi, John Tower

**Affiliations:** ^1^ Molecular and Computational Biology Section, Department of Biological Sciences, Dornsife College of Letters, Arts, and Sciences, University of Southern California, Los Angeles, CA, United States; ^2^ Buck Institute for Research on Aging, Novato, CA, United States; ^3^ Davis School of Gerontology, University of Southern California, University Park, Los Angeles, CA, United States; ^4^ Department of Environmental and Occupational Health Sciences, University of Washington, Seattle, WA, United States; ^5^ Department of Biology, University of Washington, Seattle, WA, United States; ^6^ Department of Laboratory Medicine and Pathology, University of Washington School of Medicine, Seattle, WA, United States

**Keywords:** aging, mifepristone, life span, high-fat diet, steroid, tryptophan, detoxification, hypertrophy

## Abstract

**Background:** The synthetic steroid mifepristone is reported to have anti-obesity and anti-diabetic effects in mammals on normal and high-fat diets (HFD). We previously reported that mifepristone blocks the negative effect on life span caused by mating in female *Drosophila melanogaster*.

**Methods:** Here we asked if mifepristone could protect virgin females from the life span-shortening effect of HFD. Mifepristone was assayed for effects on life span in virgin females, in repeated assays, on regular media and on media supplemented with coconut oil (HFD). The excrement quantification (EX-Q) assay was used to measure food intake of the flies after 12 days mifepristone treatment. In addition, experiments were conducted to compare the effects of mifepristone in virgin and mated females, and to identify candidate mifepristone targets and mechanisms.

**Results:** Mifepristone increased life span of virgin females on regular media, as well as on media supplemented with either 2.5 or 5% coconut oil. Food intake was not reduced in any assay, and was significantly increased by mifepristone in half of the assays. To ask if mifepristone might rescue virgin females from all life span-shortening stresses, the oxidative stressor paraquat was tested, and mifepristone produced little to no rescue. Analysis of extant metabolomics and transcriptomics data suggested similarities between effects of mifepristone in virgin and mated females, including reduced tryptophan breakdown and similarities to dietary restriction. Bioinformatics analysis identified candidate mifepristone targets, including transcription factors Paired and Extra-extra. In addition to shortening life span, mating also causes midgut hypertrophy and activation of the lipid metabolism regulatory factor SREBP. Mifepristone blocked the increase in midgut size caused by mating, but did not detectably affect midgut size in virgins. Finally, mating increased activity of a SREBP reporter in abdominal tissues, as expected, but reporter activity was not detectably reduced by mifepristone in either mated or virgin females.

**Conclusion:** Mifepristone increases life span of virgin females on regular and HFD without reducing food intake. Metabolomics and transcriptomics analyses suggest some similar effects of mifepristone between virgin and mated females, however reduced midgut size was observed only in mated females. The results are discussed regarding possible mifepristone mechanisms and targets.

## Introduction

Mifepristone (RU486) is a synthetic steroid that antagonizes the activities of human glucocorticoids and progesterone by binding to type II glucocorticoid receptor and progestin receptor, enabling it’s use as a treatment for Cushing’s disease and for birth control (Baulieu, 1997; Chen et al., 2014). Several studies also report anti-obesity and anti-diabetic effects of mifepristone in humans and mice (Gross et al., 2010; Bernal-Sore et al., 2018). For example, mifepristone improved insulin sensitivity and adiponectin levels in mice fed a high-fat diet (HFD), and caused adiponectin release from cultured adipocytes that was dependent upon PPARγ (Hashimoto et al., 2013). Consistent with those observations, mifepristone is a mammalian PPARγ agonist that activates expression of PPARγ target genes (Lin et al., 2012; Wu et al., 2018).

**FIGURE 1:**
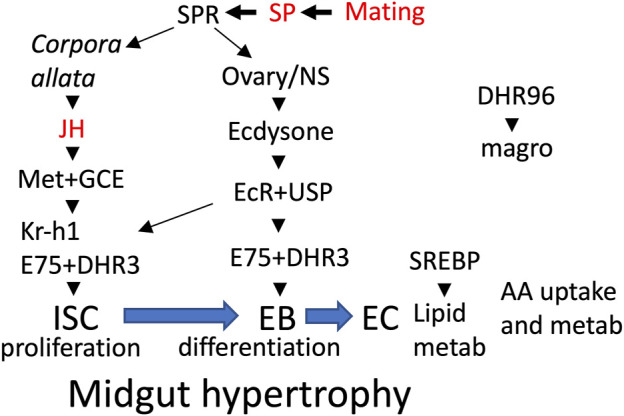
Midgut remodeling overview. Mating and male SP activate SP receptor (SPR) in multiple tissues (*Corpora allata*, ovary, nervous system), resulting in increased production of the hormones JH and ecdysone. In the midgut, JH acts through its receptors Met+GCE to activate proliferation of intestinal stem cells (ISC) and the production of their progeny the enteroblasts (EB). Also in midgut, ecdysone acts through its receptor EcR and cofactor USP to increase expression of the hormone receptor (HR) gene E75, and activation of transcription factor Kr-h1, which in turn promotes ISC proliferation and differentiation of EBs to enterocytes (EC). In the ECs, transcription factor SREBP activates genes for lipid metabolism and uptake. In proventriculus cells, the hormone receptor DHR96 activates expression of the magro lipase, which is released into the midgut lumen. Mifepristone has been shown to block the life-span shortening effects of mating, SP and the JH-analog methoprene (indicated in red font).

In female *Drosophila*, mating and male Sex Peptide (SP) hormone cause increased production of the lipid-derived juvenile hormone (JH) and the steroid hormone ecdysone (Bownes and Rembold, 1987; Bilen et al., 2013). JH and ecdysone in turn induce intestinal stem cell (ISC) proliferation, midgut growth, SREBP activation, and increased lipid metabolism (Ahmed et al., 2020; Zipper et al., 2020; White et al., 2021) (summarized in Figure 1), which supports increased egg production (Reiff et al., 2015; Sieber and Spradling, 2015). Mating and SP also cause inflammation and decreased life span, and these changes were found to be reversed by feeding the mated females the drug mifepristone, (Landis et al., 2015; Tower et al., 2017). Aging in *Drosophila* is reported to involve further ISC proliferation and mis-differentiation (“dysplasia”), regulated in part by oxidative stress, EGFR signaling, and the microbiome (Zhang et al., 2019; Zwick et al., 2019; Morris and Jasper, 2021). Mifepristone was first used in *Drosophila* as an activating trigger for the Gene-Switch artificial transcription factor (Osterwalder et al., 2001; Roman et al., 2001; Ford et al., 2007), however, the ability of mifepristone to reduce inflammation and increase life span in the mated female does not require the presence of Gene-Switch (Landis et al., 2015; Tower et al., 2017). The ability of mifepristone to increase life span appears to be female-specific, as no increase was observed in males (Landis et al., 2015).

Analysis of females mated to SP null-mutant males showed that all of the observed effects of mating on inflammation and life span required male SP, and that mifepristone acts in females to block these effects of male SP, yielding average +106% increase in median life span (Tower et al., 2017). Consistent with that result, transgenic expression of SP in virgin females recapitulated the negative effect of mating on life span, and this was also blocked by mifepristone, yielding +50 to +130% increase in median life span (Tower et al., 2017). Mifepristone did not compete with dietary ecdysone for activation of an ecdysone-responsive reporter, indicating that mifepristone does not act by directly inhibiting the ecdysone receptor (EcR), however it remains possible that mifepristone might compete with ecdysone for binding to some other ecdysone-regulated factor(s). Feeding virgin female flies the JH analog methoprene shortens life span (Flatt et al., 2005; Yamamoto et al., 2013; Landis et al., 2021), and this was also blocked by co-feeding mifepristone, yielding +63 to +90% increase in life span, and indicating that mifepristone acts to directly or indirectly antagonize JH signaling (Landis et al., 2021). Transcriptomics and targeted metabolomics were used to ask what changes are caused by mating, and reversed by mifepristone (Landis et al., 2021). Metabolites positively correlated with life span included muscle breakdown product 1/3-methylhistidine and purine breakdown product urate, suggesting possible changes in tissue turnover. Genes that were upregulated by mating, and downregulated by mifepristone were negatively correlated with life span. These genes were preferentially expressed in the midgut, and involved in protein degradation, AA metabolism and immune response. The ability of mifepristone to antagonize the negative effects of JH analog methoprene, and to inhibit expression of midgut metabolic genes, suggested the possibility that mifepristone might prevent some aspects of mating-induced midgut remodeling.

In addition to dramatically increasing the life span of mated females, mifepristone also caused smaller, but significant increases in virgin life span (average +16%) (Tower et al., 2017). Here the effects of mifepristone on virgin life span are further analyzed using normal and HFD, and the effects of mifepristone in virgins and mated females are compared using physiological markers and extant transcriptomic and targeted metabolomics data. The purpose of this study was three-fold: First was to compare the effects of mifepristone between virgin and mated female *Drosophila*. Second was to ask if there were additional stresses that could be applied to virgin females to shorten life span, and which, like mating, could be rescued by mifepristone. HFD was chosen because previous studies have shown that mifepristone can rescue some negative effects of HFD in mice (Hashimoto et al., 2013), and it was of interest to ask if mifepristone might also counteract negative effects of HFD in *Drosophila*. Paraquat was chosen because it has a unique mechanism of toxicity, involving NADPH enzyme targeting and oxidative stress (Bonneh-Barkay et al., 2005; Cocheme and Murphy, 2008), and we wished to ask if mifepristone would rescue all types of toxic stress, or whether its effects would be limited to certain stresses. The third purpose was to identify promising candidates for the targets and mechanisms for mifepristone, which could then be tested in the future.

## Methods

### *Drosophila* Strains, Culture, Life Span Assay, and Food-Intake Assay

*Drosophila melanogaster* were cultured at 25°C using a standard agar/dextrose/corn meal/yeast media (Ren et al., 2009), and adult flies were passaged to fresh media every-other day. HFD was generated by supplementing warm liquid media with the indicated concentration of coconut oil (organic triple-filtered coconut oil, Trader Joes® brand), mixing 2 min with a hand-held motorized blender, and aliquoting to vials prior to cooling. *Drosophila* strains are as previously described (Landis et al., 2021), and several were obtained from the Bloomington *Drosophila* Stock Center, including: y[1] w[*]; P{w[+mC] = elav-Switch.O}GSG301 strain (BDSC#43642, abbreviated *y; Elav-GS*), SREBP reporter strain w*; Df(2R)SCAPΔ910/CyO, P{ActGFP}JMR1; P{GAL4-dSREBPg.K}B31, P{UAS-GFP.U}3 (BDSC#39612, abbreviated SREBP reporter), and multi-copy UAS-mCherry reporter strain y[1] w[*]; PBac{y[+mDint2] w[+mC] = 20XUAS-6XmCherry-HA}VK00018/CyO, P{Wee-P.ph0}Bacc[Wee-P20]; Dr[1]/TM6C, Sb[1] Tb[1] (BDSC#52267). The *PBac{y[+mDint2] w[+mC] = 20XUAS-6XmCherry-HA}VK00018* second chromosome was re-balanced to generate genotype *y-ac-w; PBac{y[+mDint2] w[+mC] = 20XUAS-6XmCherry-HA}VK00018/CyO* (abbreviated 20XUAS-mCherry). The *w[1118]* strain is the isogenized version (*w[1118]-iso; 2-iso; 3-iso*) which was previously cured of Wolbachia by three generations treatment with doxycycline, with confirmation using PCR and Wolbachia-specific primers (Ren et al., 2007; Tower et al., 2017). To generate flies for life span assays, *w[1118]* strain males were crossed to *y; Elav-GS* strain virgin females, and hybrid female progeny were collected as virgins over 24 h. These flies were either assayed as virgins, or were mated for 48 h to young (1–2 wk of age) *w[1118]* males at a ratio of 20 males to 20 females. After mating the males were removed, and flies were maintained in culture vials in the presence/absence of drug, as indicated. Drugs were administered as previously described, by applying 100 μl of 10X stock solution in water, or 50 μl of 20X stock solution in ethanol, evenly to the surface of the vial, and allowing to absorb and dry overnight. Final concentration of drug in the media was calculated based on absorption into the top ∼1 ml of media, as determined by dye-absorption controls (Ren et al., 2009; Landis et al., 2020; Landis et al., 2021); control vials received equal volume of water or ethanol vehicle, and all vials were allowed to dry overnight. Mifepristone (RU486) was obtained from Sigma-Aldrich (cat. #M8046), and flies were treated with 200 μg/ml final concentration in the media. Paraquat (methyl viologen) was obtained from Sigma-Aldrich (cat. #856177), and flies were treated with 15 mM final concentration in the media. Median life span, percent change in median, log-rank *p* values and COX proportional hazards analyses were conducted using R statistical environment (Rcoreteam, 2020). ANOVA analyses were conducted using Prism 9. Food intake was measured using the excreta quantification (EX-Q) assay. EX-Q assay was conducted essentially as initially described (Wu et al., 2020), with minor modifications as previously described (Landis et al., 2021). Briefly, assays were conducted using 10 flies per assay chamber, and four replicate assay chambers per sample, at day 12 of drug treatment. Dye concentration in dissolved excreta was quantified relative to a standard curve using the spectrophotometer, and data is plotted as mean ± Standard Deviation in bar graphs. For all experiments, multiple comparisons were controlled using Bonferroni correction, and the corrected *p* value for significance at 5% error rate is indicated in the figure legends.

### Metabolomics and Transcriptomics Analyses

The metabolomics data is as previously described (Landis et al., 2021). In the first experiment virgin females and mated females were assayed after 12 days ± mifepristone treatment, respectively. The genotype and mating protocol were as described above for life span assays. In the second experiment, virgin females with SP overexpression (*w[1118]; UAS-SP/dsx-GAL4*) and virgin controls (*w[1118]; UAS-SP/+)* were assayed ± 12 days mifepristone treatment, respectively. Targeted metabolomics assay (∼120 metabolites) was conducted as previously described (Hoffman et al., 2014; Landis et al., 2021). Metabolites were considered significant with a false discovery rate (FDR) of 0.05 to account for multiple testing (Benjamini and Hochberg, 1995). Gene expression data is as previously described (GEO accession GSE64474), and was generated from the progeny of a cross of two transgenic strains *w[1118]; p53[B6] x w[1118]; rtTA(3)E2* (Landis et al., 2015), unrelated to the Gene-Switch system. Briefly, female progeny were collected as virgins, and half were mated to *w[1118]* strain males as described above. The virgin and mated females were maintained for 12 days ± mifepristone treatment, respectively, prior to RNA isolation and analysis as described (Landis et al., 2015).

Hypergeometric tests were carried out using online resource (https://systems.crump.ucla.edu/hypergeometric/index.php). Tissue with greatest expression level in females for selected genes was obtained from Flyatlas 2 (http://flyatlas.gla.ac.uk/FlyAtlas2/index.html). Identification of transcription factor (TF) binding sites in differentially expressed genes was conducted using publicly available ChIP-Seq data from fly larvae of 445 TFs (Negre et al., 2011; Ni et al., 2012; Slattery et al., 2014). For each TF, the enrichment of gene targets and their peak scores in an input gene set were compared to the enrichment of targets and the peak scores in 1,000,000 random draws of the same size from a background set of all *Drosophila* genes. Two permutation-corrected *p*-values for each TF were calculated, one for the number of gene targets and one for the scores of those targets. The *p*-values for peak scores were then corrected for multiple testing using both the Bonferroni and Benjamini-Hochberg methods in the python package statsmodels (Seabold and Perktold, 2010). The code for transcription factor binding site analysis is provided online (https://github.com/thilsabe/Kapahi_TF_Enrichment_Analysis.git).

### Midgut Measurements and SREBP Reporter Analysis

To generate flies for assay of maximum midgut diameter, *w[1118]* strain males were crossed to *y; Elav-GS* strain virgin females, and hybrid female progeny were collected as virgins over 24 h. Half of the flies were maintained as virgins, and half were mated for 48 h to young (1–2 wk of age) *w[1118]* males at a ratio of 20 males to 20 females. After mating the males were removed, and flies were maintained in culture vials in the presence/absence of mifepristone for 12 days. Midguts were dissected in PBS in groups of five flies at a time, and immediately mounted on slides with coverslip spaced using double-stick tape (Micchelli, 2014). Visible light images were generated and analyzed using ImageJ. The maximum diameter region of each midgut sample was estimated by inspection, multiple measurements in that region were generated using ImageJ, and the largest value was used for analysis. For analysis of SREBP reporter, the SREBP reporter strain was crossed to the 20XUAS-mCherry strain, and the hybrid females treated and dissected as described above. Both visible and red fluorescence images were generated, and overlay images are presented in the figures. Maximum midgut diameter was determined as described above. Posterior midgut diameter was determined by measuring the center of the posterior midgut region positive for mCherry expression. Quantification of posterior midgut mCherry expression was conducted using ImageJ, by selecting a region of ∼0.2 mm length of the posterior midgut, centered over the maximum of mCherry expression. For analysis of *w[1118]* strain, mated and mated plus drug samples were analyzed. Midguts were dissected in PBS, fixed for 20 min in 4% formaldehyde/PBS, washed twice in PBS, and stained for 30 min in Oil Red O solution (6 ml of 0.5% Oil Red O in isopropanol plus 4 ml demineralized water) (Luis et al., 2016). Midguts were washed twice with PBS and mounted on slides with coverslip spaced using double-stick tape, and maximum midgut diameter measured as described above. For measurements of SREBP reporter in abdomen, live flies were anesthetized using CO_2_ gas, positioned on their backs, and visible and red-fluorescence images generated. Overlay images are presented in the figure. The red images were used for quantification using ImageJ, by selecting the ventral abdomen region and measuring mean red intensity level. For all midgut assays the maximum and minimum values were censored from each group to control for outliers. Unpaired, two-sided t tests were conducted using Prism 9. For all experiments multiple comparisons were controlled using Bonferroni correction, and the corrected *p* value for significance at 5% error rate is indicated in the figure legends.

## Results

### Mifepristone Increases Life Span of Virgin Females on HFD

**FIGURE 2:**
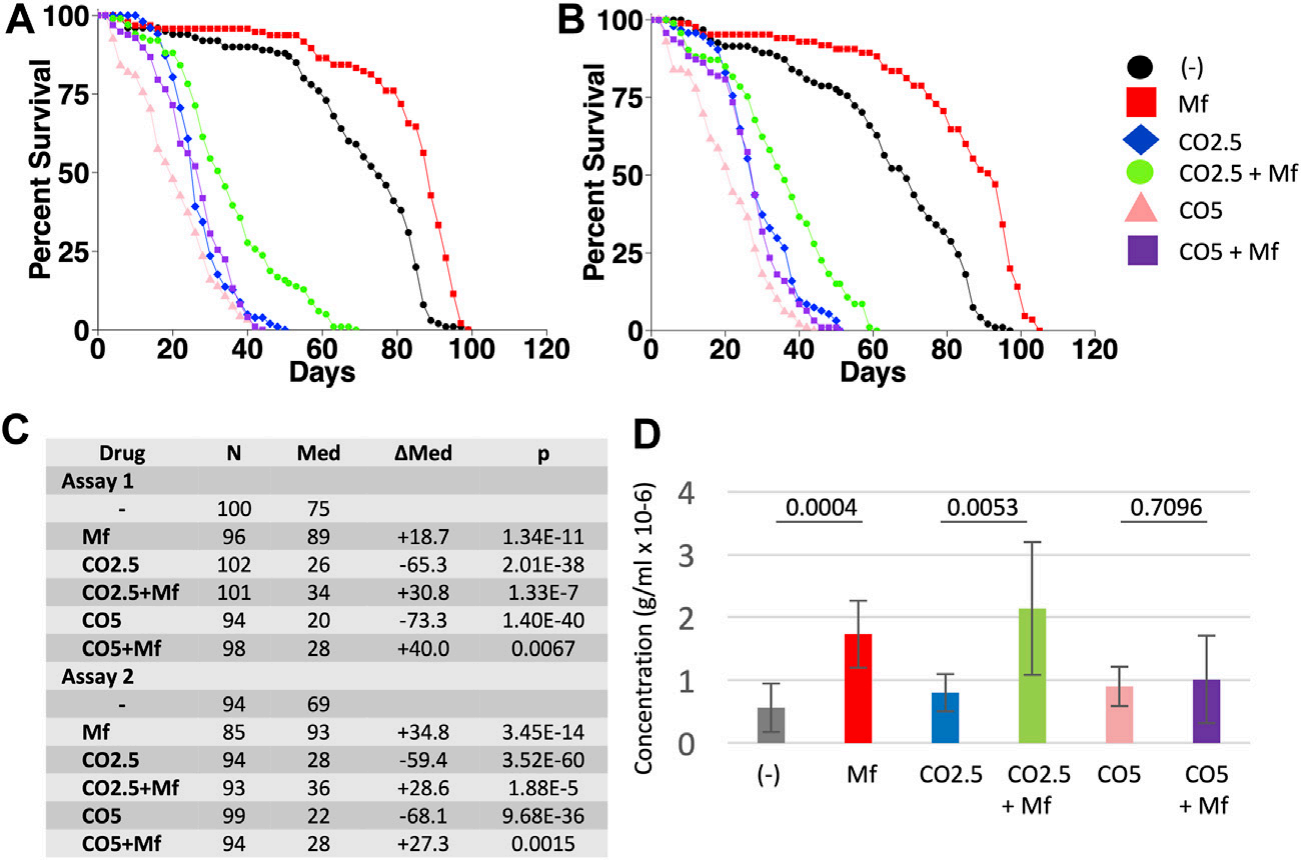
Effect of mifepristone on life span of virgin females on control and high-fat diets. Virgin females were assayed for life span on control media, media supplemented with 2.5% coconut oil (CO2.5), and media supplemented with 5% coconut oil (CO5), in the absence (−) and presence (Mf) of 200 μg/ml mifepristone. **(A)** Assay 1. **(B)** Assay 2. **(C)** Statistical summary for assays 1 and 2, including median life span and log-rank test results. Control media is compared to CO2.5 and to CO5. For each media type, (−) drug is compared to mifepristone-treated (Mf). The *p* value for significance with five comparisons is 0.01. COX proportional hazards analyses are presented in Supplementary Table S1. **(D)** Food intake determined using EX-Q assay. Data is combined for assays 1 and 2, and is presented as mean ± SD of eight replicates of 10 flies each. For each media type, (−) drug is compared to mifepristone-treated group (Mf) using unpaired, two-sided *t* test, and *p* values are presented above the bars. The *p* value for significance with one comparison is 0.05. Data for the individual assays is presented in Supplementary Figure S1, and ANOVA analyses for the individual and combined assays is presented in Supplementary Table S2.

**FIGURE 3:**
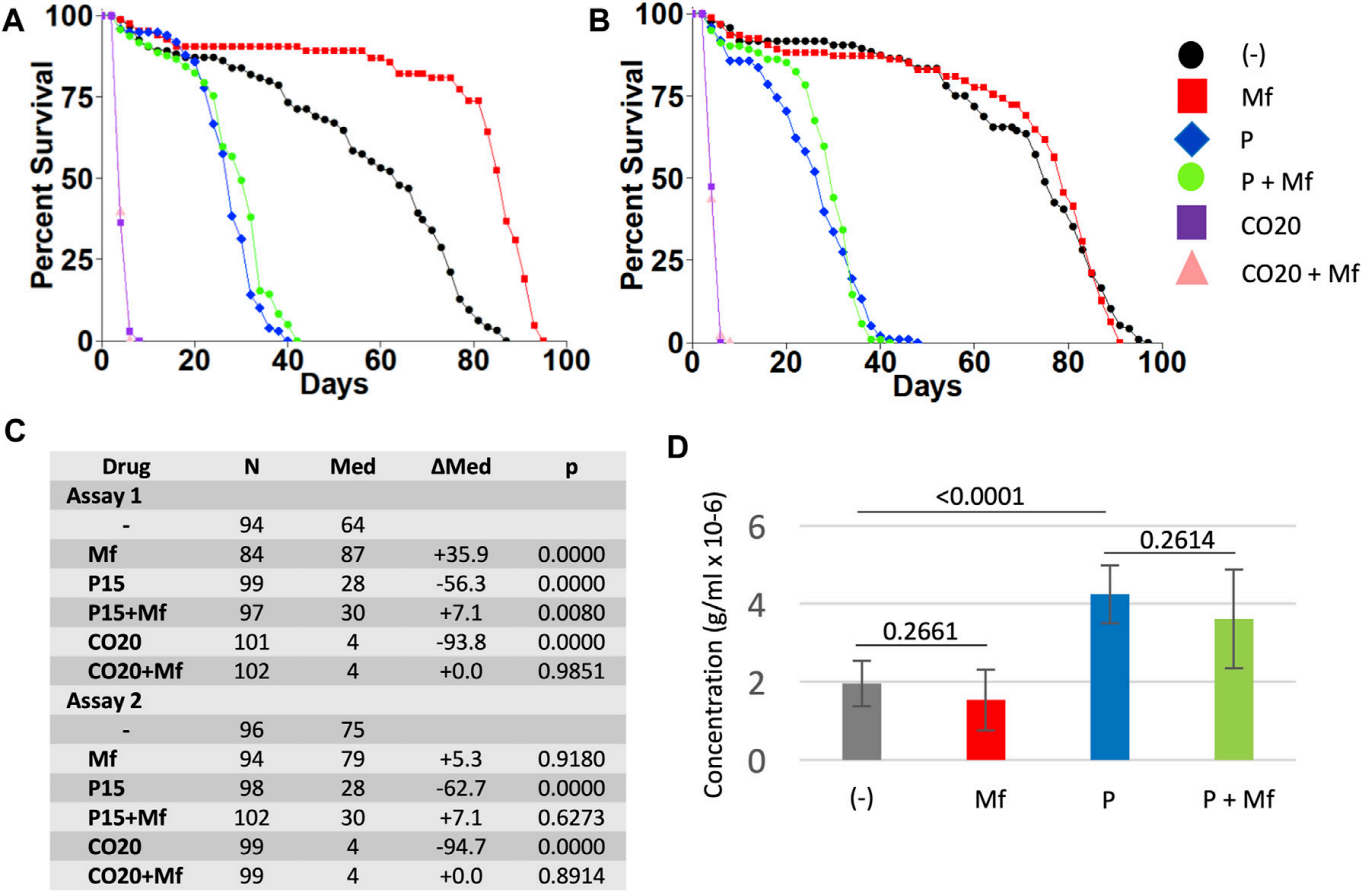
Effect of paraquat on virgin female life span in the presence and absence of mifepristone treatment, and effect of mifepristone on virgin females on 20% coconut oil media. Virgin females were assayed for life span on control media, media supplemented to 15 mM paraquat (P), and media supplemented with 20% coconut oil (CO20), in the absence (−) and presence (Mf) of 200 μg/ml mifepristone. **(A)** Assay 1. **(B)** Assay 2. **(C)** Statistical summary for assays 1 and 2, including median life span and log-rank test results. Control media is compared to P and to CO20. For each media type, (−) drug is compared to mifepristone-treated (Mf). The *p* value for significance with five comparisons is 0.01. COX proportional hazards analyses are presented in Supplementary Table S1. **(D)** Food intake determined using EX-Q assay. Data is combined for assays 1 and 2, and is presented as mean ± SD of eight replicates of 10 flies each. EX-Q assay was not feasible for CO20 flies due to limited survival. For each media type, (−) drug is compared to mifepristone-treated group (Mf) using unpaired, two-sided *t* test, and *p* values are presented above the bars. The *p* value for significance with one comparison is 0.05. Data for the individual assays is presented in Supplementary Figure S1, and ANOVA analyses for the individual and combined assays is presented in Supplementary Table S2.

In previous studies, mifepristone was found to increase the median life span of *Drosophila* mated females by average +106%, and virgin females by average +16% (Tower et al., 2017). Here the effects of mifepristone on virgin female life span were further analyzed. Virgin female flies were assayed for life span and food consumption when cultured on regular media, media supplemented to 2.5% CO, and media supplemented to 5% CO, in replicate assays. Mifepristone increased median life span on regular media by +18.7% and +34.8%, respectively (Figures 2A–C), while more than doubling food intake measured at day 12 (Figure 2D). Media with 2.5% CO reduced median life by −65.3% and −59.4%, respectively (Figures 2A–C), and mifepristone increased life span by +30.8% and +28.6%, respectively, while more than doubling food intake (Figure 2D). Media with 5% CO reduced median life by −73.3% and −68.1%, respectively (Figures 2A–C), and mifepristone increased life span by +30.8% and +28.6%, respectively, while food intake remained unchanged (Figure 2D). COX proportional hazards analysis showed a significant life span effect for mifepristone, for CO, and for the mifepristone:CO interaction, for both assays as well as the combined data (Supplementary Table S1). ANOVA for the combined EX-Q assays confirmed significant effect of mifepristone for control media and the 2.5% CO media, and the lack of effect on 5% CO media (Supplementary Table S2). These results show that mifepristone can increase life span of virgin females on HFD without decreasing food intake. Having observed that mifepristone could increase life span on media adjusted to 2.5% CO and 5% CO, it was of interest to ask if mifepristone might rescue life span at even greater concentration CO. Media supplemented to 20% CO was rapidly lethal to virgin females, thereby reducing median life span by ∼94%, and no rescue by mifepristone was observed under these conditions (Figures 3A–C).

### Mifepristone Shows Limited or No Ability to Rescue the Negative Effect of Paraquat

Virgin female flies were assayed for life span and food consumption when cultured on regular media, and media supplemented to 15 mM paraquat, in replicate assays (Figure 3). Paraquat reduced virgin female life span by −56.3% and −62.7%, respectively, and mifepristone increased life span of paraquat treated flies by +7.1% in each case, which was marginally significant in assay 1 and non-significant in assay 2, using log-rank tests. Consistent with this result, COX proportional hazards analysis showed a significant effect of mifepristone in assay 1, but not in assay 2 (Supplementary Table S1). However, COX proportional hazards analysis of the combined life span data for assays 1 and 2 showed a significant effect of paraquat, a significant effect of mifepristone, and a significant paraquat:mifepristone interaction (Supplementary Table S1). We conclude that mifepristone shows limited or no ability to rescue the negative effects of paraquat on life span. Mifepristone did not significantly alter food intake in the paraquat treated flies (Figure 3D). The effect of mifepristone on virgin female life span varied between the replicate assays in this set of experiments. Mifepristone increased life span by +35.9% in the first assay, and by +5.3% (non-significant) in the second assay (Figures 3A–C). Notably, mifepristone did not significantly alter food intake in virgin flies in these experiments (Figure 3D, Supplementary Figures S1C,D), consistent with the conclusion that mifepristone variably shows either no change in food intake or increased food intake in virgin females. Interestingly, paraquat treatment was associated with a significant increase in food intake (Figure 3D, Supplementary Figures S1C,D). ANOVA for the combined EX-Q food intake assays confirmed significant effect of paraquat, no significant effect of mifepristone, and no significant paraquat:mifepristone interaction (Supplementary Table S2).

To further confirm the ability of mifepristone to increase life span of virgin females on normal diet, two additional assays were conducted (Supplementary Figure S2). Mifepristone increased virgin female life span by +31.3% in the first assay and by +30.3% in the second assay, and significantly increased food intake in both assays. In total, mifepristone significantly increased virgin female life span on regular diet in 5/6 assays. Food intake was not decreased by mifepristone in any assay using regular or HFD, and was significantly increased in 5/10 assays.

### Analysis of Extant Metabolomics and Transcriptomics Data Indicates Similarities Between Mifepristone Effects in Virgin and Mated Females

**FIGURE 4:**
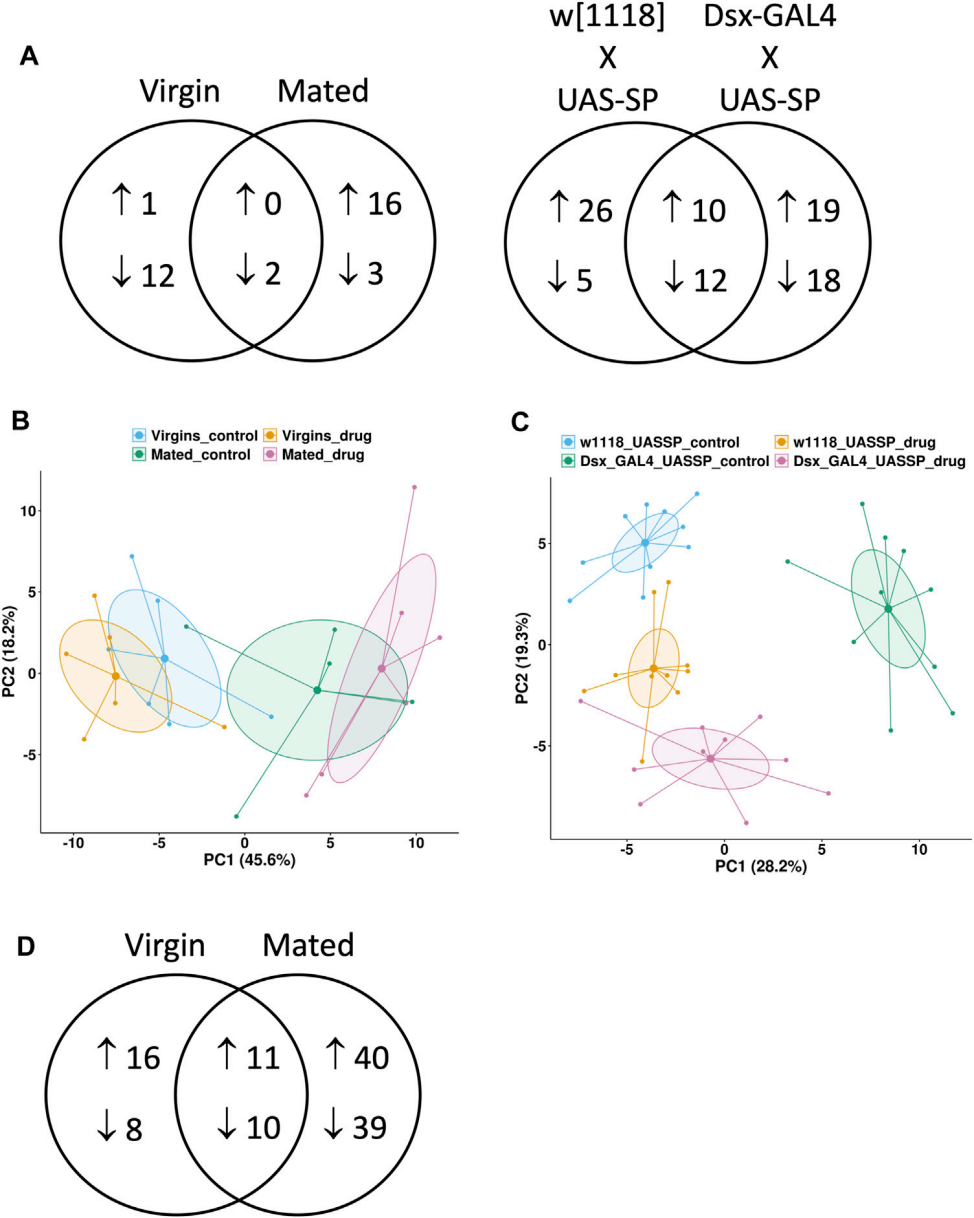
Metabolomics and transcriptomics comparisons. Targeted metabolomics assay was conducted in the presence and absence of 12 days mifepristone treatment for several types of flies. Virgin females “Virgin” and mated females “Mated” were progeny of cross *w[1118] x yw; Elav-GS*. Also assayed were virgin female progeny of two additional crosses: “*w[1118] x UAS-SP*” is the negative control for transgenic expression of SP, and “*Dsx-GAL4 x UAS-SP*” results in constitutive transgenic expression of SP. **(A)** Venn diagram showing metabolites increased and decreased by mifepristone for each type of fly. **(B**,**C)** Principal component analysis (PCA) plots of metabolite changes caused by mifepristone. Groups are indicated by colored points and 95% confidence ellipses around each group mean point at the center. The percentage of the variance explained by each PC is shown in parentheses. **(B)** PCA plot of “Virgin” females in absence (blue) and presence (yellow) of mifepristone, and “Mated” females in absence (green) and presence (pink) of mifepristone. **(C)** PCA plot of “*w[1118]* x *UAS-SP*” females in absence (blue) and presence (yellow) of mifepristone, and “*Dsx-GAL4* x *UAS-SP*” females in absence (green) and presence (pink) of mifepristone. **(D)** Venn diagram showing genes increased and decreased by mifepristone in virgin and mated females.

Targeted metabolomics analysis was conducted to compare virgin and mated females, and to compare virgin females with transgenic expression of SP to their matched virgin controls. A relatively small number of metabolite changes caused by mifepristone were detected in virgin and mated (Figure 4A, Supplementary Table S3), and principal component analysis (PCA) indicates limited divergence of the (−) mifepristone and (+) mifepristone patterns (Figure 4B). Mifepristone caused two metabolite changes in common between virgin and mated, which was down-regulation of hydroxyproline/aminolevulinate and L-kynurenine. The analysis does not distinguish between the AA hydroxyproline, and the non-proteinogenic amino acid aminolevulinate, the latter of which is a precursor to heme biosynthesis. A larger number of metabolites were altered by mifepristone in transgenic SP over-expression flies and in their controls (Figure 4A, Supplementary Table S3). PCA indicates a shift in the metabolite patterns for SP over-expression flies and controls. Based on PC1, mifepristone appears to abrogate the effects of SP overexpression. However, there is a slight increase in the effect of SP overexpression in the presence of mifepristone along the second principal component (Figure 4C). The 12 common downregulated metabolites included several AAs and lipids (Supplementary Table S3). The 10 common upregulated metabolites included ones involved in purine metabolism and glycolysis (Supplementary Table S3).

Transcriptomic analysis of effects of mifepristone in virgin and mated flies revealed 11 common up-regulated genes, including six cytochrome p450 genes, and the ABC-type xenobiotic transporter Mdr50, consistent with upregulation of drug detoxification pathways (Figure 4D, Supplementary Table S4). The 10 common downregulated genes included BomBc1, which is a secreted anti-microbial peptide, and Lsp1beta, a larval storage protein that may support adult protein synthesis (Supplementary Table S4). Also downregulated were eight genes involved in synthesis of the chorion (eggshell). The gene expression changes caused by mifepristone in virgin and mated females were compared to several published studies of gene expression changes cause by various stresses and environmental effects (Table 1). For both virgin females and mated females, the pattern of changes caused by mifepristone was most similar to changes caused by DR, by rapamycin treatment, and by sleep deprivation (Zimmerman et al., 2006; Dobson et al., 2018).

**TABLE 1:** Effect of mifepristone in mated and virgin females compared to various stresses.

Stress		Ref	Sex	Total	Mf	Stress	Overlap	*p*	Result
Virgin vs stress									
	Landis aging	Landis et al. (2012)	M	13,964	45	1470	15	3.25–5	over 3.17
	Landis O2	Landis et al. (2012)	M	13,964	45	897	12	1.83–5	over 4.15
	Landis H2O2	Landis et al. (2012)	M	13,964	45	1641	11	0.0132	over 2.08
	Landis heat	Landis et al. (2012)	M	13,964	45	1566	16	1.56–5	over 3.17
	Landis IR 907 Gy	Landis et al. (2012)	M	13,964	45	1388	9	0.0306	over 2.01
	Moskalev cold 4°	Moskalev et al. (2015)	M	13,964	45	869	10	0.0004	over 3.57
	Moskalev IR 144 Gy	Moskalev et al. (2015)	M	13,964	45	195	3	0.0248	over 4.77
	Moskalev starvation	Moskalev et al. (2015)	M	13,964	45	79	2	0.0267	over 7.86
	Dobson DR	Dobson et al. (2018)	F	13,964	45	442	17	1.16E−14	over 11.94
	Dobson rapa	Dobson et al. (2018)	F	13,964	45	350	14	2.46E−12	over 12.41
	Gershman yeast feeding	Gershman et al. (2007)	F	13,964	45	631	4	0.1445	over 1.97
	Mack mating	Mack et al. (2006)	F	13,964	45	545	3	0.2562	over 1.71
	Wang tubule expression	Wang et al. (2004)	ND	13,964	45	1315	5	0.4197	over 1.1.8
	King-Jones PB	King-Jones et al. (2006)	ND	13,964	45	828	17	2.70E−10	over 6.37
	King-Jones PB DHR96 mutant	King-Jones et al. (2006)	ND	13,964	45	503	9	2.65E−5	over 5.55
	Zimmerman sleep (Table 1)	Zimmerman et al. (2006)	F	13,964	45	44	4	1.16E−5	over 28.21
	Deng rox deficiency males	Deng and Meller, (2006)	M	13,964	45	3464	20	0.0031	over 1.79
Mated vs stress									
	Landis aging	Landis et al. (2012)	M	13,964	100	1470	27	2.95E−6	over 2.56
	Landis O2	Landis et al. (2012)	M	13,964	100	897	27	9.24E−11	over 4.2
	Landis H2O2	Landis et al. (2012)	M	13,964	100	1641	29	2.452E−6	over 2.47
	Landis heat	Landis et al. (2012)	M	13,964	100	1566	31	7.52E−8	over 2.76
	Landis IR 907 Gy	Landis et al. (2012)	M	13,964	100	1388	21	0.0007	over 2.11
	Moskalev cold 4°	Moskalev et al. (2015)	M	13,964	100	869	34	5.93E−17	over 5.46
	Moskalev IR 144 Gy	Moskalev et al. (2015)	M	13,964	100	195	9	1.07E−5	over 6.44
	Moskalev starvation	Moskalev et al. (2015)	M	13,964	100	79	1	0.4341	over 1.77
	Dobson DR	Dobson et al. (2018)	F	13,964	100	442	39	9.65E−33	over 12.32
	Dobson rapa	Dobson et al. (2018)	F	13,964	100	350	34	9.78E−30	over 13.57
	Gershman yeast feeding	Gershman et al. (2007)	F	13,964	100	631	7	0.1662	over 1.55
	Mack mating	Mack et al. (2006)	F	13,964	100	545	2	0.2458	under 1.95
	Wang tubule expression	Wang et al. (2004)	ND	13,964	100	1315	15	0.0471	over 1.59
	King-Jones PB	King-Jones et al. (2006)	ND	13,964	100	828	35	1.54E−10	over 5.9
	King-Jones PB DHR96 mutant	King-Jones et al. (2006)	ND	13,964	100	503	17	9.25E−8	over 4.72
	Zimmerman sleep (Table 1)	Zimmerman et al. (2006)	F	13,964	100	44	4	0.0003	over 12.69
	Deng rox deficiency	Deng and Meller, (2006)	M	13,964	100	3464	42	0.0001	over 1.69

The total number of genes altered by mifepristone (Mf) in virgin and mated females is compared to the number of genes altered by various stresses in either female or male flies. Hypergeometric test, *p* value for significance with 17 comparisons is 0.0029. Cutoff for fold change is 1.5. Result column is the over- or under-representation of genes in the overlap relative to the number of genes expected by chance. DR, dietary restriction; Rapa, rapamycin; PB, phenobarbital; ND, not defined.

The genes regulated by mifepristone were analyzed for possible transcription factor (TF) binding site enrichment using a custom script written in python. The analysis utilized a publicly available set of TF binding sites identified in *Drosophila* larval tissues using ChIP-seq (Negre et al., 2011; Ni et al., 2012; Slattery et al., 2014). The enrichment of gene targets and their peak scores in an input gene set of 445 TFs was compared to the enrichment of targets and peak scores in 1,000,000 random draws of the same gene set size from a background set of all *Drosophila* genes. Two permutation-corrected *p*-values for each TF were calculated, one for the number of gene targets and one for the scores of those targets. The *p*-values for peak scores were then corrected for multiple testing using both the Bonferroni and Benjamini-Hochberg methods in the python package statsmodels (Seabold and Perktold, 2010). The genes regulated by mifepristone in mated females were enriched for binding sites for the transcription factors Paired (prd) and Extra-extra (exex) (Table 2). Paired and Extra-extra were also the top hits for binding sites in genes upregulated by mating. Analysis of genes regulated by mifepristone in virgins also identified Paired and Extra-extra, however this was not significant after controls for multiple comparisons (Table 2).

**TABLE 2:** Transcription factor binding site enrichment in genes regulated by mating and by mifepristone.

Comparison	TF	CG#	Total TF genes	TF genes in set	Number p	Score p	B-H corrected *p*	Bonferroni corrected *p*
Mated (+) vs mated (−)								
	prd	CG6716	5546	77	0	0	0	0
	exex	CG8254	4109	57	0	0.000013	0.0028925	0.005785
Mated vs Virgin (downregulated)								
	psq	CG2368	4128	28	0.000061	0.000001	0.000297	0.000445
	Sin3A	CG8815	3025	31	0	0.000002	0.000297	0.00089
	Stat92E	CG4257	9349	43	0.002365	0.000002	0.000297	0.00089
	YL-1	CG4621	4548	38	0	0.000004	0.000445	0.00178
	gfzf	CG33546	3981	34	0	0.000008	0.000712	0.00356
	Sry-delta	CG17958	4632	34	0.000006	0.00001	0.000742	0.00445
	M1BP	CG9797	3749	38	0	0.000017	0.001081	0.007565
	HmgD	CG17950	2478	26	0	0.000021	0.001168	0.009345
	CG4617	CG4617	4508	33	0.000019	0.000024	0.001187	0.01068
	Crg-1	CG32788	2862	28	0.000001	0.000034	0.001513	0.01513
	Dif	CG6794	2629	22	0.00005	0.000042	0.001699	0.01869
	Clamp	CG1832	1980	18	0.000044	0.000054	0.001985	0.02403
	CG1620	CG1620	3122	29	0	0.000058	0.001985	0.02581
	ovo	CG6824	3098	26	0.000015	0.000067	0.00213	0.029815
Mated vs Virgin (upregulated)								
	exex	CG8254	4109	311	0	0	0	0
	prd	CG6716	5546	373	0	0	0	0
	grn	CG9656	3627	234	0.000005	0.000015	0.002225	0.006675
Virgin (+) vs Virgin (−)								
	prd	CG6716	5546	21	0.007	0.012	NS	NS
	exex	CG8254	4109	18	0.014	0.07	NS	NS
	cic	CG43122	3909	14	0.16	0.447	NS	NS
	cnc	CG43286	695	3	0.278	0.316	NS	NS

(+) and (−) refer to mifepristone treatment.

### Mifepristone Reduces Midgut Size in Mated Females

**FIGURE 5:**
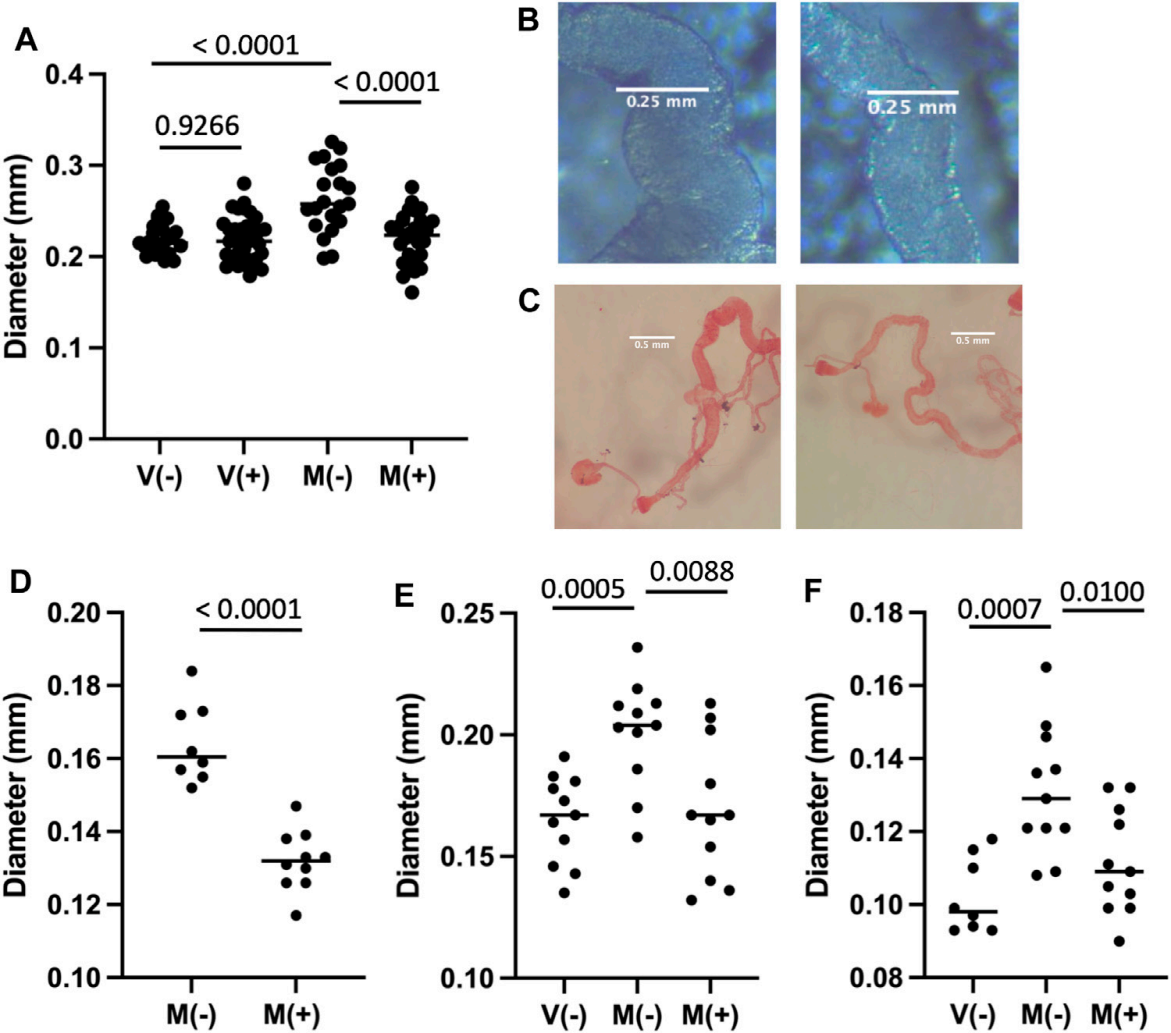
Effect of mifepristone on midgut size. The midgut was dissected from virgin females (V) or mated females (M), maintained for 12 days in the absence (−) or presence (+) of 200 μg/ml mifepristone, as indicated. Midgut tissue was dissected, mounted on slides, photographed, and the maximum midgut diameter was measured using ImageJ. Statistical comparisons are unpaired, two-sided *t* test, and *p* values are presented above the groups. **(A**,**B)** Progeny of cross *w[1118] x yw;Elav-GS*. **(A)** Maximum midgut diameter. The *p* value for significance with three comparisons is 0.0167. **(B)** Examples of midgut middle region. Left panel M(−), right panel M(+). **(C**,**D)** *w[1118]* strain. **(C)** Examples of midgut, fixed and stained with Oil Red O dye. Left panel M(−), right panel M(+). **(D)** Maximum *w[1118]* strain midgut diameter quantification. The *p* value for significance with one comparison is 0.05. **(E**,**F)** Progeny of cross *SREBP-R x UAS-mCherry*. **(E)** Maximum midgut diameter. **(F)** PM diameter. The *p* value for significance with two comparisons is 0.025.

Mating and male SP hormone are known to induce JH and ecdysone hormone signaling pathways that in turn cause metabolic remodeling, ISC proliferation and hypertrophy in the female midgut (Reiff et al., 2015; Ahmed et al., 2020; Zipper et al., 2020; White et al., 2021) (Figure 1). Flies of the genotype used above for life span assays (progeny of *w[1118]* x *yw; Elav-GS* cross) were assayed for maximum midgut diameter in virgin females and mated females, in the presence and absence of 12 days mifepristone treatment, respectively (Figures 5A,B). Mating caused increased maximum midgut diameter, as expected, and mifepristone treatment prevented this increase. In contrast, mifepristone did not detectably alter midgut size in virgin females (Figure 5A). Reduction of mated female midgut size by mifepristone was confirmed in two additional genotypes, *w[1118]* (Figure 5D) and SREBP reporter flies (Figures 5E,F).

### Mifepristone Does Not Significantly Reduce Activation of a SREBP Reporter

**FIGURE 6:**
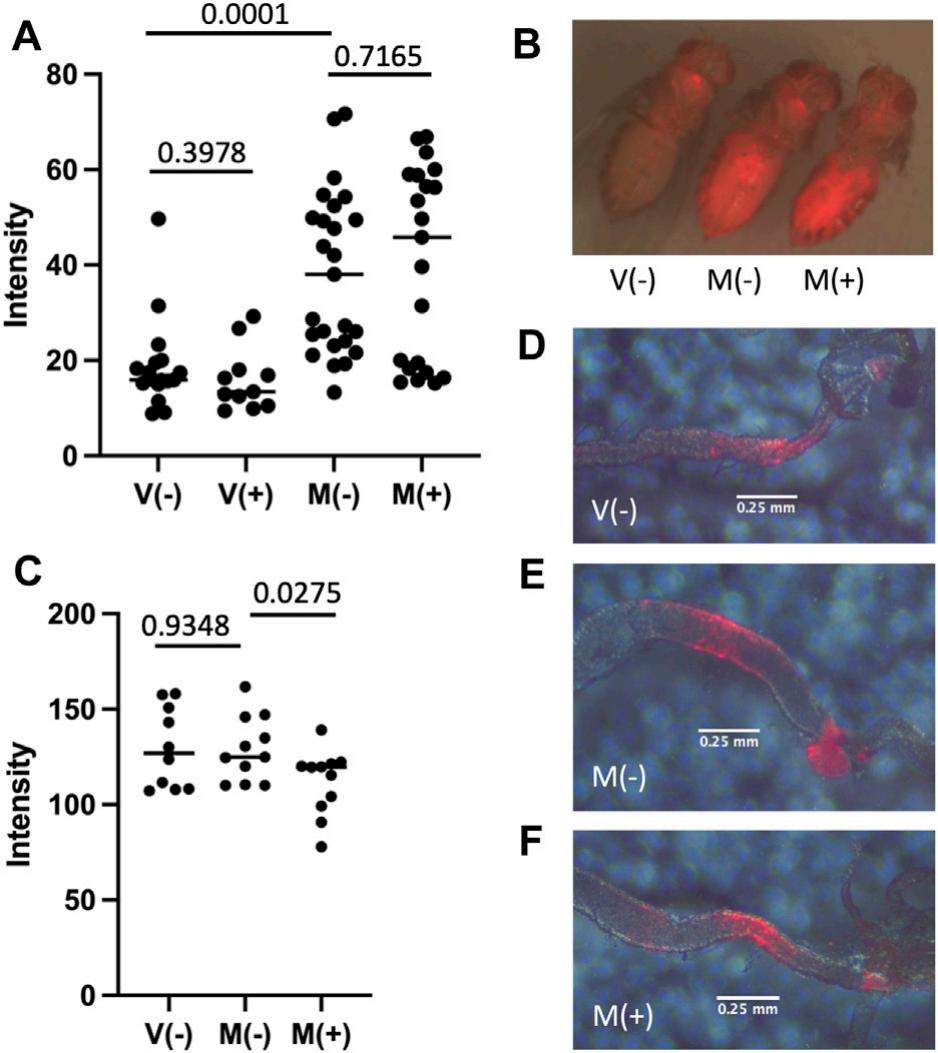
Effect of mifepristone on SREBP reporter activity. Flies are the progeny of cross *SREBP-R x UAS-mCherry*. Virgin females (V) and mated females (M) were maintained for 12 days in the absence (−) or presence (+) of 200 μg/ml mifepristone, as indicated. **(A**,**B)** Florescence intensity of the abdomen was quantified using red fluorescence image capture and ImageJ. **(A)** Abdominal fluorescence quantification. **(B)** Representative flies. Image presented is overlay of red fluorescence image and visible light images. **(C)** Posterior midgut fluorescence quantification. Midgut tissue was dissected, mounted on slides, and florescence intensity of the posterior midgut was quantified using red fluorescence image capture and ImageJ. **(D**–**F)** Representative posterior midguts. Image presented is overlay of red fluorescence image and visible light images. Errors bars are placed below the region of the posterior midgut where the SREBP reporter activates expression of mCherry. **(D)** V(−). **(E)** M(−). **(F)** M(+).

Sterol regulatory element-binding protein (SREBP) is synthesized as an ER membrane protein and then transported to the Golgi where it is activated by proteolytic processing. The active SREBP transcription factor then translocates to the nucleus where it activates genes required for lipid biosynthesis (Rawson, 2003). The SREBP reporter is a fusion of the regulatory region of the *Drosophila* SREBP protein, the yeast GAL4 DNA binding domain, and the herpes simplex virus protein VP16 transcriptional activation domain (Kunte et al., 2006). The fusion protein undergoes the activating proteolytic processing of the SREBP moiety to produce an active transcription factor that binds to UAS sites in the promoter of engineered target constructs. The SREBP reporter strain was crossed to the UAS-mCherry target strain such that activation of SREBP reporter protein produces expression of mCherry. Virgin flies (V) and mated flies (M) were maintained in the absence (−) or presence (+) of mifepristone for 12 days, and then assayed for mCherry fluorescence in the abdomen (Figures 6A,B), and in dissected posterior midgut tissue (Figures 6C–F). Mating caused increased mean reporter activity in the abdomen, including apparent fat-body, oenocyte and gut tissue expression, and this was not detectably altered by mifepristone treatment (Figures 6A,B). Interestingly, the activation of SREBP reporter by mating suggests a possible bi-phasic pattern, where some flies show relatively high-level induction and others show little or no induction. The pattern of SREBP reporter expression in the adult gut is reported to include a localized region of expression in the posterior midgut that is increased by mating (Reiff et al., 2015; Zipper et al., 2020). Dissection and analysis of posterior midgut region indicated no detectable increase in mean expression level for mCherry due to mating, and no significant decrease due to mifepristone treatment after control for multiple comparisons (Figure 6C). Analysis of the diameter of the posterior midgut region exhibiting SREBP reporter expression confirmed increased posterior midgut size upon mating, and prevention of this size increase by mifepristone (Figure 5F).

## Discussion

### Mifepristone Increases Life Span of Virgin Females on HFD Without Decreasing Food Intake

The experiments presented here show that mifepristone increases median life span of virgin females on a HFD containing either 2.5% CO or 5% CO, without decreasing food intake. In addition, mifepristone increased life span of virgin females on normal diet in three out of four assays. At the same time mifepristone increased virgin life span, it was often associated with increased food intake, including two out of four assays on normal media, and both assays of 2.5% CO media. These results are consistent with our previous observations that mifepristone often increases mated female food intake at the same time it increases median life span (Landis et al., 2015; Tower et al., 2017). Similarly, feeding virgin female flies the JH analog methoprene shortened life span, and co-feeding mifepristone partly rescued the life span decrease, while at the same time doubling food intake (Landis et al., 2021). We speculate that a decrease in some aspect of virgin female metabolism caused by mifepristone signals the fly to compensate by increasing food intake (Landis et al., 2021). For example, inhibiting fatty-acid beta-oxidation increases food intake in both *C. elegans* and mammals (Horn et al., 2004; Bouagnon et al., 2019). Media supplemented to 20% CO was highly toxic, and all flies were dead within 6 days. No rescue by mifepristone was observed under these conditions, and one possible interpretation is that the flies were unable to survive long enough for the mifepristone to exert any beneficial physiological effects. In the future, it may be of interest to ask if pre-treatment of the flies with mifepristone might provide some protection from 20% CO.

Mifepristone showed limited or no ability to rescue the negative effect of the oxidative stressor paraquat on virgin female life span, indicating that mifepristone does not rescue all toxic stresses. This result is consistent with the observation that there is a lack of sensitive oxidative stress-response genes, such as *Hsp70*, *Hsp22* and *GstD1/2* (Sykiotis and Bohmann, 2008; Landis et al., 2012), among the genes regulated by mifepristone. Also, the genes regulated by mifepristone in both virgin and mated females showed greater overlap with genes regulated by DR, rapamycin and sleep disruption than they did with genes regulated by oxidative stress (Table 1). The conclusion that mifepristone does not rescue all toxic stresses is also supported by our previous observation that feeding octopamine decreased virgin female life span, and co-feeding mifepristone did not rescue this effect, but instead made the life span decrease more severe (Landis et al., 2021). Mating has been reported to increase starvation resistance and to decrease oxidative stress resistance in female *Drosophila* (Rush et al., 2007). In the future, it may be of interest to ask if mifepristone might prevent these changes, concordant with its ability to prevent midgut hypertrophy, inflammation and decreased life span.

### Metabolomics Analysis Suggest Mifepristone Inhibits Tryptophan Breakdown in Virgin and Mated Females

Analysis of metabolomic data revealed some similarities between the effects of mifepristone in virgin and mated females, and between virgin females with transgenic expression of SP and their controls. Mifepristone caused decreased levels of hydroxyproline/aminolevulinate and L-kynurenine in both virgin and mated females. Kynurenine is a breakdown product of tryptophan, indicating that mifepristone inhibits tryptophan breakdown in both virgin and mated females (Supplementary Table S3). In virgin females with transgenic expression of SP and their matched virgin controls, mifepristone caused decreased levels of the AAs asparagine, glutamic acid, glutamine, lysine and threonine, and the AA precursor homoserine, indicating downregulation of AA metabolism and/or uptake (Supplementary Table S3). Also downregulated in both groups were several lipids, including 13-HODE, margaric acid, and myristic acid, as well as the ketone body 3HBA and the TCA cycle intermediate oxaloacetate (Supplementary Table S3). Finally, kynurenine was decreased by mifepristone in virgin females with transgenic expression of SP, and tryptophan breakdown product kynurenic acid was decreased by mifepristone in the matched controls, again indicating decreased breakdown of tryptophan. Taken together, the data indicate that the shared effects of mifepristone in virgin and mated females include decreased AA and lipid metabolism, and in particular, decreased tryptophan breakdown.

Tryptophan breakdown is of particular interest, as it has previously been implicated in aging and life span regulation in several ways. For example, inhibition of tryptophan breakdown is reported to suppress the proteotoxicity of aggregation-prone proteins and extend life span in *C. elegans* (van der Goot et al., 2012). There is some information on role of tryptophan breakdown in regulating *Drosophila* life span. The *vermilion* gene encodes the Trp-2,3-dioxygenase (TDO) enzyme, which catalyzes tryptophan conversion into kynurenine, and in a *Canton-S* genetic background the *vermilion* mutation was reported to decrease male life span and have no effect on female life span (Navrotskaya and Oxenkrug, 2016). In contrast, an inhibitor of TDO (alpha-methyl tryptophan) and an inhibitor of ABC transporters implicated in tryptophan import (5-methyl tryptophan) were reported to increase adult female life span (Oxenkrug et al., 2011). In mammals, levels of circulating kynurenine increase with age, and have been linked to increased mortality, osteoporosis, inflammation and neurological diseases, whereas kynurenic acid has been reported to be neuroprotective (Breda et al., 2016; Hamrick and Isales, 2020). In addition, the gut microbiota is reported to modulate host immune function by modulating tryptophan metabolism, through mechanisms involving activation of the aryl hydrocarbon receptor (AhR) by tryptophan metabolites (Gao et al., 2018). Investigating the possibility that mifepristone increases female *Drosophila* life span by reducing tryptophan breakdown may be a productive area for future research.

There were no common metabolites upregulated by mifepristone between virgin and mated groups, however there were several in common between virgin females with transgenic expression of SP and their controls. The metabolites upregulated by mifepristone included three involved in purine metabolism (8-Oxo-2′-deoxyguanosine, cGMP, and inosine), and one involved in nucleotide sugar metabolism UDP-GlcNAc. Also upregulated by mifepristone was a hexose phosphate sugar involved in glycolysis (the assay does not distinguish between Glucose1P/Glucose6P/Fructose1P/Fructose6P), and the glycolysis product lactate (Supplementary Table S3). In the future, it may be of interest to assay the effects of mifepristone on life span when additional components of the diet are adjusted, including carbohydrates, total proteins, and individual AAs such as tryptophan.

### Transcriptomics Analysis Indicates Mifepristone Activates Detoxification Genes in Virgin and Mated Females and has Similarities to DR

Analysis of extant transcriptomics data also indicated similarities between the effects of mifepristone in virgin and mated females. The 10 common downregulated genes included BomBc1, which is a secreted anti-microbial peptide (Supplementary Table S4). Downregulation of innate immune response genes by mifepristone was previously observed for mated females. For example, the anti-microbial peptide gene *Drosocin* is upregulated by mating and downregulated by mifepristone, and this pattern of regulation was confirmed using a transgenic reporter construct (Tower et al., 2017). Also downregulated in both virgin and mated females was Lsp1beta, which encodes a larval storage protein. Decreased synthesis of Lsp1beta protein in the adult might be related to the decrease in AA metabolism indicated by the metabolomics changes discussed above. Also among the common genes downregulated in virgin and mated females were eight genes involved in synthesis of the chorion (eggshell). This result is consistent with the previous observation that mifepristone reduces progeny production in mated females (Landis et al., 2015), and suggests the intriguing possibility that choriogenesis or chorion gene expression might be costly for life span. However, female progeny of *Tudor* mutant females lack a germline and produce no eggs with eggshells, and yet have a normal life span, which argues against a role for choriogenesis (Barnes et al., 2006; Shen et al., 2009).

There were 11 genes up-regulated by mifepristone in both virgin and mated females. These included six cytochrome p450 genes reported to be involved in insecticide detoxification, and the ABC-type xenobiotic transporter Mdr50, indicating upregulation of drug detoxification pathways (Supplementary Table S4). Consistent with this observation, there was significant overlap between genes altered by mifepristone and genes reported to be altered by feeding the drug phenobarbital (Table 2). The upregulation of drug detoxification genes in both virgin and mated females in response to mifepristone suggests a possible “cross-detoxification” type mechanism, in which the enzymes induced to detoxify mifepristone also degrade some endogenous compounds that would otherwise reduce life span, such as steroid hormones or other life-span limiting metabolites.

Comparison of the gene expression changes caused by mifepristone to gene expression changes reported in other studies shows the greatest matches to DR, rapamycin treatment, and sleep disruption (Table 1). The similarity to DR is consistent with the reduced midgut size in mated females, which might be expected to decrease nutrient absorption. If mifepristone also reduces nutrient absorption in virgin females, this would be consistent with the partial rescue of life span on HFD, and might explain the frequent observations of increased food consumption as a compensatory response of the fly to the reduced nutrient uptake. The similarity to rapamycin treatment is of particular interest, as rapamycin inhibits the TOR signaling pathway (Bjedov and Rallis, 2020). TOR signaling promotes protein synthesis and growth, limits life span in *Drosophila* and other organisms, and decreased TOR signaling is implicated in the mechanism of DR (Kapahi et al., 2010; Kapahi et al., 2016; Bjedov and Rallis, 2020). If mifepristone directly or indirectly inhibits TOR signaling this would be consistent with the inhibitory effects of mifepristone on AA metabolism and midgut hypertrophy. The similarity between the gene expression changes caused by mifepristone and by sleep disruption is also of interest, particularly given recent reports linking *Drosophila* sleep, circadian rhythms and gut function (Katewa et al., 2016; Parasram et al., 2018; Ulgherait et al., 2020; Vaccaro et al., 2020).

### Mifepristone Reduces Midgut Size in Mated Females

Mating and SP have been reported to act through JH and ecdysone signaling pathways to induce the metabolic remodeling and hypertrophy of the midgut (Reiff et al., 2015; Ahmed et al., 2020; Zipper et al., 2020) (Figure 1). Consistent with those results, feeding adult virgin females the JH analog methoprene induces midgut metabolic remodeling and hypertrophy and shortens life span (Yamamoto et al., 2013; Reiff et al., 2015). We previously reported that mifepristone can partly rescue the negative effect of methoprene on virgin life span, indicating that mifepristone counteracts JH signaling to increase life span, and suggesting the hypothesis that mifepristone may be acting to increase life span by inhibiting midgut metabolic remodeling and/or hypertrophy. Here we confirm that mating causes an increase in female midgut size, and find that indeed mifepristone prevents this increase in midgut size in the mated female. The decreased midgut size in the mated female will reduce the surface area available for absorption and the volume of cells available to carry out midgut metabolism, and is therefore expected to reduce nutrient absorption and total midgut metabolism. This may contribute to the life span extension caused by mifepristone in the mated female, and is consistent with the similarities to DR discussed above. As shown here and in previous studies, mifepristone also increases life span in virgin females in most assays. However, mifepristone did not detectably decrease midgut size in the virgin female. These results suggest that either there is a decrease in midgut size in virgin females that is too small to detect, or perhaps more likely, the mechanisms for life span increase involve effects in addition to inhibiting midgut hypertrophy, such as more direct inhibition of absorptive and/or metabolic pathways.

### Mifepristone Did Not Detectably Inhibit Activation of a SREBP Reporter

SREBP is a conserved transcriptional regulator of genes involved in lipid uptake and metabolism (Rawson, 2003). Mean intensity of SREBP reporter expression in the female fly abdomen was doubled by mating, consistent with previous reports that mating induces SREBP (Reiff et al., 2015; Sieber and Spradling, 2015; Zipper et al., 2020). It is of potential interest that the activation of SREBP reporter by mating suggests a possible bi-phasic pattern, where some flies show relatively high-level induction and others show little or no induction (Figure 6A). This suggests a possible all-or-none type activation of SREBP by mating at the level of the individual female fly. Inspection of the abdomen indicated increased SREBP expression in fat-body, oenocytes and gut tissues (Figure 6B). Mean intensity of expression of the SREBP reporter in dissected posterior midgut tissue was not detectably increased by mating (Figure 6C). However, because the posterior midgut increases in size in response to mating, and decreases in size in response to mifepristone (Figure 5F), this means there is variation in the number and/or volume of posterior midgut cells expressing SREBP, and therefore variation in the amount of total posterior midgut SREBP activity at the level of the individual fly. Mifepristone did not detectably decrease total SREBP reporter expression in abdomen, and while there was a trend towards decreased activity in dissected posterior midgut tissue, this was not significant after control for multiple comparisons (Figure 6C). These results suggest that mifepristone does not act by direct inhibition of SREBP activation, however the reduction in size of midgut tissue expressing SREBP may still be a contributing factor to the life span increase caused by mifepristone in mated females. Recently, Ma et al. reported that mifepristone inhibited Estrogen-related receptor (ERR) reporter activity in adult *Drosophila*, and reduced lipid content and magro lipase gene expression in the adult midgut, suggesting the possibility that ERR might be a mifepristone target (Ma et al., 2021). However, fly sex was not defined in that study, so it is not clear if the observed effects occurred in males or females, or might reflect variation in the ratio of male and female flies in the samples.

### Candidates for Mifepristone Targets

The results presented here suggest several candidates for factors that might mediate the effects of mifepristone on life span. Because mifepristone is a synthetic steroid that antagonizes steroid hormone receptors in mammals, one possibility is that mifepristone might antagonize steroid hormone signaling in *Drosophila*. Mifepristone did not detectably inhibit the activation of an ecdysone-responsive reporter by dietary ecdysone, indicating that mifepristone does not directly antagonize the ecdysone receptor (EcR) (Landis et al., 2021), however it remains possible that mifepristone might interact with some other factor(s) normally regulated by ecdysone or other hormones. One candidate target for mifepristone is the hormone receptor Eip75B (75B), which is reported to regulate midgut hypertrophy in mated females (Ahmed et al., 2020; Zipper et al., 2020) (Figure 1). Eip75B is related to mammalian PPARγ, and mammalian PPARγ is reported to bind mifepristone (Lin et al., 2012), suggesting the possibility that mifepristone might inhibit midgut remodeling by binding to Eip75B. Analysis of transcription factor binding sites that are enriched in the promoter regions of genes regulated by mating and regulated by mifepristone in mated females identified the transcription factors Paired (prd) and Extra-extra (exex) (Table 2). Analysis of genes regulated by mifepristone in virgins also identified Paired and Extra-extra, however this was not significant after controls for multiple comparisons. Paired is an essential transcription factor with two DNA binding domains, a paired domain and a homeodomain. Paired regulates embryonic segmentation, including development of the abdomen and the larval midgut (Dohrmann et al., 1990; Harbecke and Lengyel, 1995). Extra-extra is a homeodomain transcription factor that regulates differentiation of developing neurons, including insulinergic neurons that innervate the hindgut (Miguel-Aliaga et al., 2008). Extra-extra is expressed in enteroendocrine (EE) cells in the central region of the adult female midgut, and is required in these cells for expression of the peptide hormone orcokinin (Guo et al., 2019). In the future, it may be of interest to experimentally modulate the activity of the candidate mifepristone targets and life span regulators identified here, including Eip75B, Paired, Extra-extra, ERR, tryptophan pathway enzymes and detoxification genes, and assay for effects on life span in response to mating, mifepristone and HFD.

### Potential Relevance to Human Health

Sex differences in human aging-associated diseases are common, and understanding sex differences will be important in designing effective interventions (Ngo et al., 2014; Kautzky-Willer et al., 2016; Kim et al., 2018; Tower et al., 2020). The ability of mifepristone to increase life span of virgin females on HFD reported here further extends the similarities between mifepristone effects in flies and mammals. Mifepristone improved insulin sensitivity and adiponectin levels in mice fed a HFD (Hashimoto et al., 2013), and mifepristone is reported to have anti-diabetes and anti-obesity effects in humans and mice (Gross et al., 2010; Bernal-Sore et al., 2018; Diaz-Castro et al., 2020; Gubbi et al., 2021). The female sex-specificity of mifepristone life span effects in *Drosophila*, and the implication of midgut as a relevant target, also suggests some similarities to human health. For example, women have greater prevalence of obesity and gastrointestinal (GI) disorders than men, including GI disorders associated with pregnancy (Prusator and Chang, 2017). In several mammalian species, lactation is associated with remodeling and growth of the intestine to support increased digestion and nutrient uptake (Hammond, 1997). In rat intestine, lactation was associated with upregulation of SREBP target genes involved in lipid uptake and metabolism (Athippozhy et al., 2011). In the future, it may be of interest to ask if mifepristone might decrease mammalian gut size and/or metabolism, similar to its effects in female *Drosophila*.

## Conclusion

The ability of mifepristone to prevent the mating-induced increase in midgut size in *Drosophila* mated females suggests this may be one mechanism for the dramatic life span extension (∼+100%) caused by mifepristone in the mated female. The ability of mifepristone to increase life span in virgin female *Drosophila* on HFD (∼+30%) suggests that mifepristone may have additional targets and mechanisms. Some of these mifepristone mechanisms in *Drosophila* might be relevant to mammals and the beneficial effects of mifepristone on mammalian metabolic phenotypes.

## Data Availability

The datasets presented in this study can be found in online repositories. The names of the repository/repositories and accession number(s) can be found in the article/Supplementary Material.
