# Supplementary figures and images for: Mifepristone Increases Life Span of Virgin Female Drosophila on Regular and High-fat Diet Without Reducing Food Intake
Source: Front Genet. 2021 Sep 24;12:751647. doi: 10.3389/fgene.2021.751647 (PMC8511958; doi:10.3389/fgene.2021.751647)

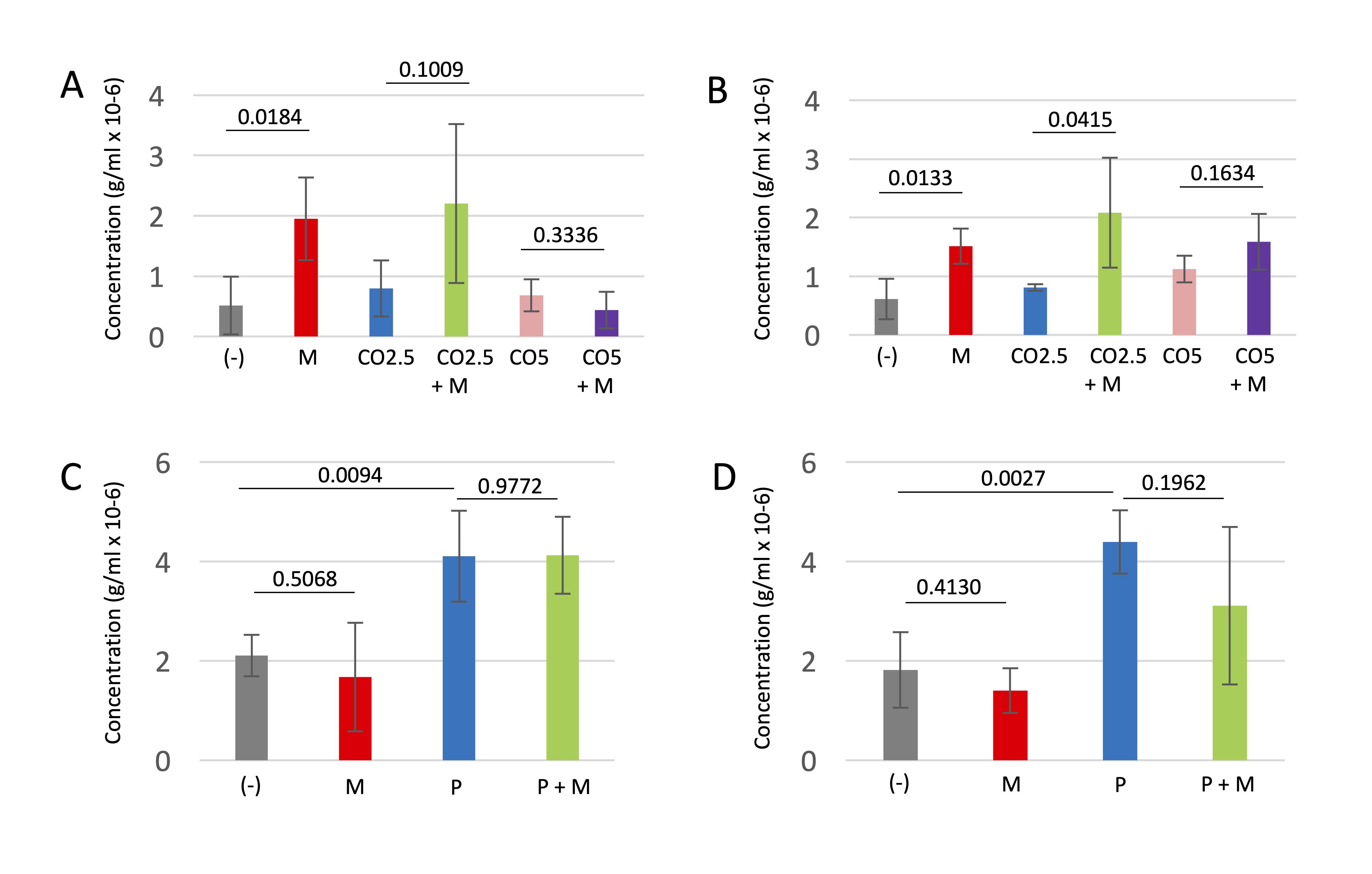

Supplement: Supplementary file 1 [file Image1.TIFF]

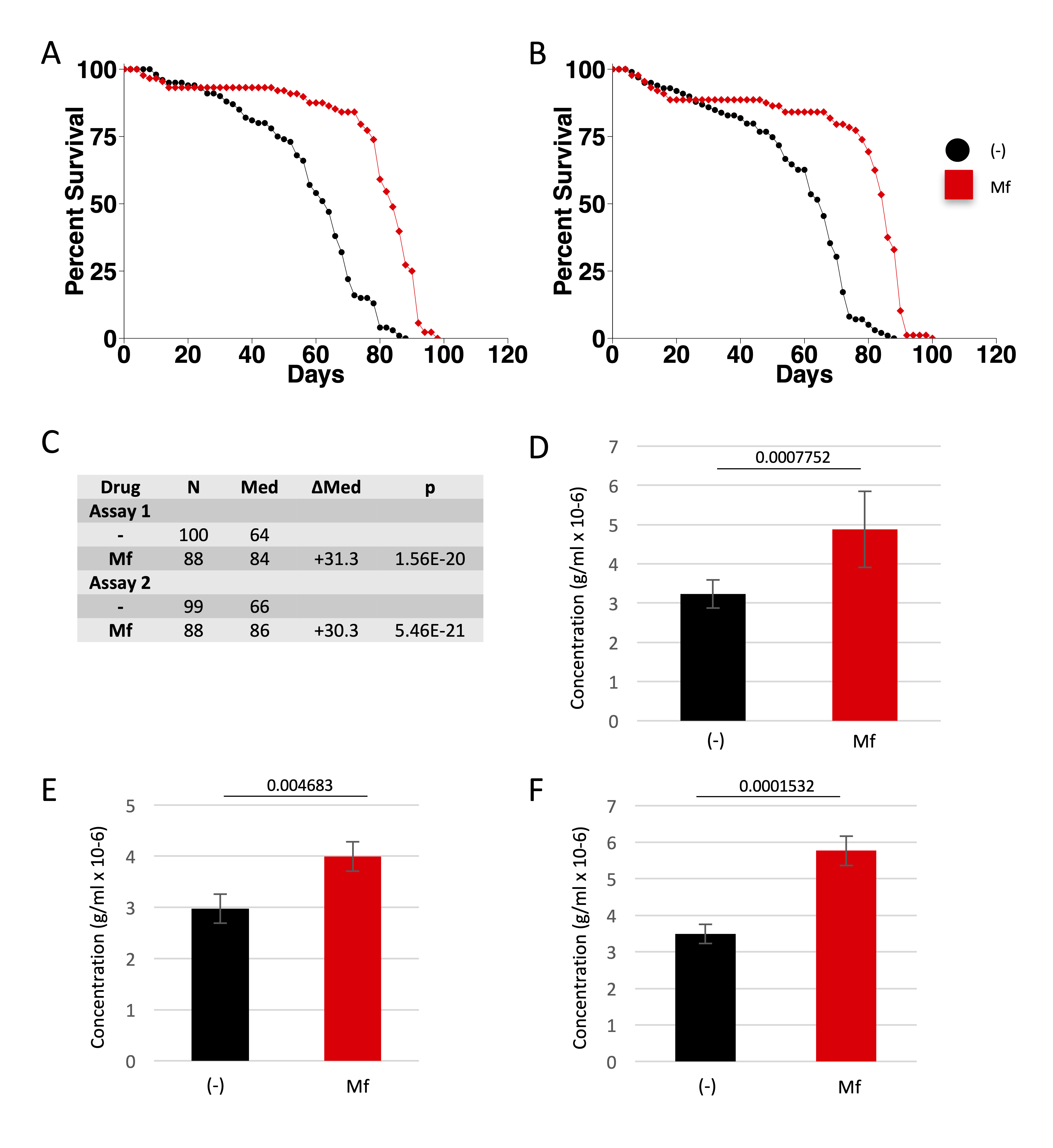

Supplement: Supplementary file 6 [file Image2.TIFF]
