# Supplementary material for: Mifepristone Increases Life Span of Virgin Female Drosophila on Regular and High-fat Diet Without Reducing Food Intake
Source: Front Genet. 2021 Sep 24;12:751647. doi: 10.3389/fgene.2021.751647 (PMC8511958; doi:10.3389/fgene.2021.751647)
Supplement: Supplementary file 4 [file DataSheet5.pdf]

Supplemental Figure legends

Figure S1. Food intake assayed using EX-Q for individual experiments. Data is presented as mean +/-SD of 4 replicates of 10 flies each. For each media type, (-) drug is compared to mifepristone-treated group (M) using unpaired, two-sided t test, and p values are presented above the bars. The p value for significance with one comparison is 0.05. **(A, B)** Data for individual EX-Q assays of Figure 1 **(A)** Assay 1. **(B)** Assay 2. **(C, D)** Data for individual EX-Q assays of Figure 2. **(C)** Assay 1. **(B)** Assay 2. ANOVA analyses for the individual and combined assays is presented in Supplementary Table S2.

Figure S2. Effect of mifepristone on life span of virgin females on control diet, additional assays. Virgin females were assayed for life span on control media, in the absence (-) and presence (Mf) of 200μg/ml mifepristone. **(A)** Assay 1. **(B)** Assay 2. **(C)** Statistical summary for assays 1 and 2, including median life span and log-rank test results. (-) drug is compared to mifepristone-treated (Mf). The p value for significance with 1 comparisons is 0.05. **(D-F)** Food intake determined using EX-Q assay. (-) drug is compared to mifepristone-treated group (Mf) using unpaired, two-sided t test, and p values are presented above the bars. The p value for significance with one comparison is 0.05. (**D**) Combined data for assay 1 and assay 2. (**E**) Assay 1. **(F)** Assay 2.
