# Supplementary material for: Mifepristone Increases Life Span of Virgin Female Drosophila on Regular and High-fat Diet Without Reducing Food Intake
Source: Front Genet. 2021 Sep 24;12:751647. doi: 10.3389/fgene.2021.751647 (PMC8511958; doi:10.3389/fgene.2021.751647)
Supplement: Supplementary file 5 [file DataSheet2.docx]

**Table S2. ANOVA for EX-Q assays**

**Figure 2. CO Assay 1. 2-way ANOVA, Rows are CO, Columns are RU486**

| Table Analyzed | Grouped: Two-way ANOVA (two data sets) |  |  |  |  |
| --- | --- | --- | --- | --- | --- |
|  |  |  |  |  |  |
| Two-way ANOVA | Ordinary |  |  |  |  |
| Alpha | 0.05 |  |  |  |  |
|  |  |  |  |  |  |
| Source of Variation | % of total variation | P value | P value summary | Significant? |  |
| Interaction | 17.34 | 0.0502 | ns | No |  |
| Row Factor | 17.57 | 0.0486 | * | Yes |  |
| Column Factor | 21.11 | 0.0088 | ** | Yes |  |
|  |  |  |  |  |  |
| ANOVA table | SS | DF | MS | F (DFn, DFd) | P value |
| Interaction | 0.07940 | 2 | 0.03970 | F (2, 18) = 3.549 | P=0.0502 |
| Row Factor | 0.08042 | 2 | 0.04021 | F (2, 18) = 3.595 | P=0.0486 |
| Column Factor | 0.09665 | 1 | 0.09665 | F (1, 18) = 8.641 | P=0.0088 |
| Residual | 0.2013 | 18 | 0.01118 |  |  |
|  |  |  |  |  |  |
| Difference between column means |  |  |  |  |  |
| Mean of -RU486 | 0.1036 |  |  |  |  |
| Mean of +RU486 | 0.2305 |  |  |  |  |
| Difference between means | -0.1269 |  |  |  |  |
| SE of difference | 0.04318 |  |  |  |  |
| 95% CI of difference | -0.2176 to -0.03621 |  |  |  |  |
|  |  |  |  |  |  |
| Data summary |  |  |  |  |  |
| Number of columns (Column Factor) | 2 |  |  |  |  |
| Number of rows (Row Factor) | 3 |  |  |  |  |
| Number of values | 24 |  |  |  |  |

Pairwise comparisons with Bonferroni correction

| Compare each cell mean with the other cell mean in that row |  |  |  |  |  |  |  |  |
| --- | --- | --- | --- | --- | --- | --- | --- | --- |
|  |  |  |  |  |  |  |  |  |
| Number of families | 1 |  |  |  |  |  |  |  |
| Number of comparisons per family | 3 |  |  |  |  |  |  |  |
| Alpha | 0.05 |  |  |  |  |  |  |  |
|  |  |  |  |  |  |  |  |  |
| Bonferroni's multiple comparisons test | Mean Diff. | 95.00% CI of diff. | Below threshold? | Summary | Adjusted P Value |  |  |  |
|  |  |  |  |  |  |  |  |  |
| -RU486 - +RU486 |  |  |  |  |  |  |  |  |
| -CO | -0.2105 | -0.4079 to -0.01314 | Yes | * | 0.0344 |  |  |  |
| 2.5% CO | -0.2060 | -0.4034 to -0.008637 | Yes | * | 0.0391 |  |  |  |
| 5% CO | 0.03575 | -0.1616 to 0.2331 | No | ns | >0.9999 |  |  |  |
|  |  |  |  |  |  |  |  |  |
|  |  |  |  |  |  |  |  |  |
| Test details | Mean 1 | Mean 2 | Mean Diff. | SE of diff. | N1 | N2 | t | DF |
|  |  |  |  |  |  |  |  |  |
| -RU486 - +RU486 |  |  |  |  |  |  |  |  |
| -CO | 0.08150 | 0.2920 | -0.2105 | 0.07478 | 4 | 4 | 2.815 | 18.00 |
| 2.5% CO | 0.1230 | 0.3290 | -0.2060 | 0.07478 | 4 | 4 | 2.755 | 18.00 |
| 5% CO | 0.1063 | 0.07050 | 0.03575 | 0.07478 | 4 | 4 | 0.4781 | 18.00 |

**Figure 2. CO Assay 2. 2-way ANOVA, Rows are CO, Columns are RU486**

| Table Analyzed | Grouped: Two-way ANOVA (two data sets) |  |  |  |  |
| --- | --- | --- | --- | --- | --- |
|  |  |  |  |  |  |
| Two-way ANOVA | Ordinary |  |  |  |  |
| Alpha | 0.05 |  |  |  |  |
|  |  |  |  |  |  |
| Source of Variation | % of total variation | P value | P value summary | Significant? |  |
| Interaction | 6.143 | 0.3117 | ns | No |  |
| Row Factor | 6.012 | 0.3190 | ns | No |  |
| Column Factor | 43.43 | 0.0005 | *** | Yes |  |
|  |  |  |  |  |  |
| ANOVA table | SS | DF | MS | F (DFn, DFd) | P value |
| Interaction | 0.01407 | 2 | 0.007035 | F (2, 18) = 1.245 | P=0.3117 |
| Row Factor | 0.01377 | 2 | 0.006885 | F (2, 18) = 1.218 | P=0.3190 |
| Column Factor | 0.09946 | 1 | 0.09946 | F (1, 18) = 17.60 | P=0.0005 |
| Residual | 0.1017 | 18 | 0.005652 |  |  |
|  |  |  |  |  |  |
| Difference between column means |  |  |  |  |  |
| Mean of -RU486 | 0.1305 |  |  |  |  |
| Mean of +RU486 | 0.2593 |  |  |  |  |
| Difference between means | -0.1288 |  |  |  |  |
| SE of difference | 0.03069 |  |  |  |  |
| 95% CI of difference | -0.1932 to -0.06427 |  |  |  |  |
|  |  |  |  |  |  |
| Data summary |  |  |  |  |  |
| Number of columns (Column Factor) | 2 |  |  |  |  |
| Number of rows (Row Factor) | 3 |  |  |  |  |
| Number of values | 24 |  |  |  |  |
|  |  |  |  |  |  |
|  |  |  |  |  |  |

Pairwise comparisons with Bonferroni correction

| Compare each cell mean with the other cell mean in that row |  |  |  |  |  |  |  |  |
| --- | --- | --- | --- | --- | --- | --- | --- | --- |
|  |  |  |  |  |  |  |  |  |
| Number of families | 1 |  |  |  |  |  |  |  |
| Number of comparisons per family | 3 |  |  |  |  |  |  |  |
| Alpha | 0.05 |  |  |  |  |  |  |  |
|  |  |  |  |  |  |  |  |  |
| Bonferroni's multiple comparisons test | Mean Diff. | 95.00% CI of diff. | Below threshold? | Summary | Adjusted P Value |  |  |  |
|  |  |  |  |  |  |  |  |  |
| -RU486 - +RU486 |  |  |  |  |  |  |  |  |
| -CO | -0.1318 | -0.2720 to 0.008544 | No | ns | 0.0700 |  |  |  |
| 2.5% CO | -0.1865 | -0.3268 to -0.04621 | Yes | ** | 0.0075 |  |  |  |
| 5% CO | -0.06800 | -0.2083 to 0.07229 | No | ns | 0.6512 |  |  |  |
|  |  |  |  |  |  |  |  |  |
|  |  |  |  |  |  |  |  |  |
| Test details | Mean 1 | Mean 2 | Mean Diff. | SE of diff. | N1 | N2 | t | DF |
|  |  |  |  |  |  |  |  |  |
| -RU486 - +RU486 |  |  |  |  |  |  |  |  |
| -CO | 0.09600 | 0.2278 | -0.1318 | 0.05316 | 4 | 4 | 2.478 | 18.00 |
| 2.5% CO | 0.1248 | 0.3113 | -0.1865 | 0.05316 | 4 | 4 | 3.508 | 18.00 |
| 5% CO | 0.1708 | 0.2388 | -0.06800 | 0.05316 | 4 | 4 | 1.279 | 18.00 |

**Figure 2. CO Assays 1+2 combined. 2-way ANOVA, Rows are CO, Columns are RU486**

| Table Analyzed | Grouped: Two-way ANOVA (two data sets) |  |  |  |  |
| --- | --- | --- | --- | --- | --- |
|  |  |  |  |  |  |
| Two-way ANOVA | Ordinary |  |  |  |  |
| Alpha | 0.05 |  |  |  |  |
|  |  |  |  |  |  |
| Source of Variation | % of total variation | P value | P value summary | Significant? |  |
| Interaction | 10.94 | 0.0211 | * | Yes |  |
| Row Factor | 6.692 | 0.0867 | ns | No |  |
| Column Factor | 28.17 | <0.0001 | **** | Yes |  |
|  |  |  |  |  |  |
| ANOVA table | SS | DF | MS | F (DFn, DFd) | P value |
| Interaction | 0.07614 | 2 | 0.03807 | F (2, 42) = 4.237 | P=0.0211 |
| Row Factor | 0.04659 | 2 | 0.02329 | F (2, 42) = 2.593 | P=0.0867 |
| Column Factor | 0.1961 | 1 | 0.1961 | F (1, 42) = 21.83 | P<0.0001 |
| Residual | 0.3773 | 42 | 0.008984 |  |  |
|  |  |  |  |  |  |
| Difference between column means |  |  |  |  |  |
| Mean of -RU486 | 0.1170 |  |  |  |  |
| Mean of +RU486 | 0.2449 |  |  |  |  |
| Difference between means | -0.1278 |  |  |  |  |
| SE of difference | 0.02736 |  |  |  |  |
| 95% CI of difference | -0.1831 to -0.07262 |  |  |  |  |
|  |  |  |  |  |  |
| Data summary |  |  |  |  |  |
| Number of columns (Column Factor) | 2 |  |  |  |  |
| Number of rows (Row Factor) | 3 |  |  |  |  |
| Number of values | 48 |  |  |  |  |
|  |  |  |  |  |  |
|  |  |  |  |  |  |
|  |  |  |  |  |  |
|  |  |  |  |  |  |
|  |  |  |  |  |  |
|  |  |  |  |  |  |

Pairwise comparisons with Bonferroni correction

| Compare each cell mean with the other cell mean in that row |  |  |  |  |  |  |  |  |
| --- | --- | --- | --- | --- | --- | --- | --- | --- |
|  |  |  |  |  |  |  |  |  |
| Number of families | 1 |  |  |  |  |  |  |  |
| Number of comparisons per family | 3 |  |  |  |  |  |  |  |
| Alpha | 0.05 |  |  |  |  |  |  |  |
|  |  |  |  |  |  |  |  |  |
| Bonferroni's multiple comparisons test | Mean Diff. | 95.00% CI of diff. | Below threshold? | Summary | Adjusted P Value |  |  |  |
|  |  |  |  |  |  |  |  |  |
| -RU486 - +RU486 |  |  |  |  |  |  |  |  |
| -CO | -0.1711 | -0.2893 to -0.05295 | Yes | ** | 0.0024 |  |  |  |
| 2.5% CO | -0.1963 | -0.3144 to -0.07807 | Yes | *** | 0.0005 |  |  |  |
| 5% CO | -0.01613 | -0.1343 to 0.1021 | No | ns | >0.9999 |  |  |  |
|  |  |  |  |  |  |  |  |  |
|  |  |  |  |  |  |  |  |  |
| Test details | Mean 1 | Mean 2 | Mean Diff. | SE of diff. | N1 | N2 | t | DF |
|  |  |  |  |  |  |  |  |  |
| -RU486 - +RU486 |  |  |  |  |  |  |  |  |
| -CO | 0.08875 | 0.2599 | -0.1711 | 0.04739 | 8 | 8 | 3.611 | 42.00 |
| 2.5% CO | 0.1239 | 0.3201 | -0.1963 | 0.04739 | 8 | 8 | 4.141 | 42.00 |
| 5% CO | 0.1385 | 0.1546 | -0.01613 | 0.04739 | 8 | 8 | 0.3403 | 42.00 |
|  |  |  |  |  |  |  |  |  |

**Figure 3. Paraquat Assay 1. 2-way ANOVA, Rows are Paraquat, Columns are RU486**

| Table Analyzed | Grouped: Two-way ANOVA (two data sets) |  |  |  |  |
| --- | --- | --- | --- | --- | --- |
|  |  |  |  |  |  |
| Two-way ANOVA | Ordinary |  |  |  |  |
| Alpha | 0.05 |  |  |  |  |
|  |  |  |  |  |  |
| Source of Variation | % of total variation | P value | P value summary | Significant? |  |
| Interaction | 0.6911 | 0.6171 | ns | No |  |
| Row Factor | 67.24 | 0.0003 | *** | Yes |  |
| Column Factor | 0.5807 | 0.6465 | ns | No |  |
|  |  |  |  |  |  |
| ANOVA table | SS | DF | MS | F (DFn, DFd) | P value |
| Interaction | 0.004356 | 1 | 0.004356 | F (1, 12) = 0.2634 | P=0.6171 |
| Row Factor | 0.4238 | 1 | 0.4238 | F (1, 12) = 25.62 | P=0.0003 |
| Column Factor | 0.003660 | 1 | 0.003660 | F (1, 12) = 0.2213 | P=0.6465 |
| Residual | 0.1985 | 12 | 0.01654 |  |  |
|  |  |  |  |  |  |
| Difference between column means |  |  |  |  |  |
| Mean of -RU486 | 0.4610 |  |  |  |  |
| Mean of +RU486 | 0.4308 |  |  |  |  |
| Difference between means | 0.03025 |  |  |  |  |
| SE of difference | 0.06430 |  |  |  |  |
| 95% CI of difference | -0.1099 to 0.1704 |  |  |  |  |
|  |  |  |  |  |  |
| Difference between row means |  |  |  |  |  |
| Mean of -Paraquat | 0.2831 |  |  |  |  |
| Mean of +Paraquat | 0.6086 |  |  |  |  |
| Difference between means | -0.3255 |  |  |  |  |
| SE of difference | 0.06430 |  |  |  |  |
| 95% CI of difference | -0.4656 to -0.1854 |  |  |  |  |
|  |  |  |  |  |  |
| Interaction CI |  |  |  |  |  |
| Mean diff, A1 - B1 | 0.06325 |  |  |  |  |
| Mean diff, A2 - B2 | -0.002750 |  |  |  |  |
| (A1 -B1) - (A2 - B2) | 0.06600 |  |  |  |  |
| 95% CI of difference | -0.2142 to 0.3462 |  |  |  |  |
| (B1 - A1) - (B2 - A2) | -0.06600 |  |  |  |  |
| 95% CI of difference | -0.3462 to 0.2142 |  |  |  |  |
|  |  |  |  |  |  |
| Data summary |  |  |  |  |  |
| Number of columns (Column Factor) | 2 |  |  |  |  |
| Number of rows (Row Factor) | 2 |  |  |  |  |
| Number of values | 16 |  |  |  |  |

Pairwise comparisons with Bonferroni correction

| Compare each cell mean with the other cell mean in that row |  |  |  |  |  |  |  |  |
| --- | --- | --- | --- | --- | --- | --- | --- | --- |
|  |  |  |  |  |  |  |  |  |
| Number of families | 1 |  |  |  |  |  |  |  |
| Number of comparisons per family | 2 |  |  |  |  |  |  |  |
| Alpha | 0.05 |  |  |  |  |  |  |  |
|  |  |  |  |  |  |  |  |  |
| Bonferroni's multiple comparisons test | Mean Diff. | 95.00% CI of diff. | Below threshold? | Summary | Adjusted P Value |  |  |  |
|  |  |  |  |  |  |  |  |  |
| -RU486 - +RU486 |  |  |  |  |  |  |  |  |
| -Paraquat | 0.06325 | -0.1696 to 0.2961 | No | ns | >0.9999 |  |  |  |
| +Paraquat | -0.002750 | -0.2356 to 0.2301 | No | ns | >0.9999 |  |  |  |
|  |  |  |  |  |  |  |  |  |
|  |  |  |  |  |  |  |  |  |
| Test details | Mean 1 | Mean 2 | Mean Diff. | SE of diff. | N1 | N2 | t | DF |
|  |  |  |  |  |  |  |  |  |
| -RU486 - +RU486 |  |  |  |  |  |  |  |  |
| -Paraquat | 0.3148 | 0.2515 | 0.06325 | 0.09094 | 4 | 4 | 0.6955 | 12.00 |
| +Paraquat | 0.6073 | 0.6100 | -0.002750 | 0.09094 | 4 | 4 | 0.03024 | 12.00 |

**Figure 3. Paraquat Assay 2. 2-way ANOVA, Rows are Paraquat, Columns are RU486**

| Table Analyzed | Grouped: Two-way ANOVA (two data sets) |  |  |  |  |
| --- | --- | --- | --- | --- | --- |
|  |  |  |  |  |  |
| Two-way ANOVA | Ordinary |  |  |  |  |
| Alpha | 0.05 |  |  |  |  |
|  |  |  |  |  |  |
| Source of Variation | % of total variation | P value | P value summary | Significant? |  |
| Interaction | 2.214 | 0.4028 | ns | No |  |
| Row Factor | 53.97 | 0.0011 | ** | Yes |  |
| Column Factor | 8.478 | 0.1155 | ns | No |  |
|  |  |  |  |  |  |
| ANOVA table | SS | DF | MS | F (DFn, DFd) | P value |
| Interaction | 0.01613 | 1 | 0.01613 | F (1, 12) = 0.7521 | P=0.4028 |
| Row Factor | 0.3931 | 1 | 0.3931 | F (1, 12) = 18.33 | P=0.0011 |
| Column Factor | 0.06175 | 1 | 0.06175 | F (1, 12) = 2.879 | P=0.1155 |
| Residual | 0.2574 | 12 | 0.02145 |  |  |
|  |  |  |  |  |  |
| Difference between column means |  |  |  |  |  |
| Mean of -RU486 | 0.4608 |  |  |  |  |
| Mean of +RU486 | 0.3365 |  |  |  |  |
| Difference between means | 0.1243 |  |  |  |  |
| SE of difference | 0.07322 |  |  |  |  |
| 95% CI of difference | -0.03529 to 0.2838 |  |  |  |  |
|  |  |  |  |  |  |
| Difference between row means |  |  |  |  |  |
| Mean of -Paraquat | 0.2419 |  |  |  |  |
| Mean of +Paraquat | 0.5554 |  |  |  |  |
| Difference between means | -0.3135 |  |  |  |  |
| SE of difference | 0.07322 |  |  |  |  |
| 95% CI of difference | -0.4730 to -0.1540 |  |  |  |  |
|  |  |  |  |  |  |
| Interaction CI |  |  |  |  |  |
| Mean diff, A1 - B1 | 0.06075 |  |  |  |  |
| Mean diff, A2 - B2 | 0.1878 |  |  |  |  |
| (A1 -B1) - (A2 - B2) | -0.1270 |  |  |  |  |
| 95% CI of difference | -0.4461 to 0.1921 |  |  |  |  |
| (B1 - A1) - (B2 - A2) | 0.1270 |  |  |  |  |
| 95% CI of difference | -0.1921 to 0.4461 |  |  |  |  |
|  |  |  |  |  |  |
| Data summary |  |  |  |  |  |
| Number of columns (Column Factor) | 2 |  |  |  |  |
| Number of rows (Row Factor) | 2 |  |  |  |  |
| Number of values | 16 |  |  |  |  |

Pairwise comparisons with Bonferroni correction

| Compare each cell mean with the other cell mean in that row |  |  |  |  |  |  |  |  |
| --- | --- | --- | --- | --- | --- | --- | --- | --- |
|  |  |  |  |  |  |  |  |  |
| Number of families | 1 |  |  |  |  |  |  |  |
| Number of comparisons per family | 2 |  |  |  |  |  |  |  |
| Alpha | 0.05 |  |  |  |  |  |  |  |
|  |  |  |  |  |  |  |  |  |
| Bonferroni's multiple comparisons test | Mean Diff. | 95.00% CI of diff. | Below threshold? | Summary | Adjusted P Value |  |  |  |
|  |  |  |  |  |  |  |  |  |
| -RU486 - +RU486 |  |  |  |  |  |  |  |  |
| -Paraquat | 0.06075 | -0.2044 to 0.3259 | No | ns | >0.9999 |  |  |  |
| +Paraquat | 0.1878 | -0.07735 to 0.4529 | No | ns | 0.1898 |  |  |  |
|  |  |  |  |  |  |  |  |  |
|  |  |  |  |  |  |  |  |  |
| Test details | Mean 1 | Mean 2 | Mean Diff. | SE of diff. | N1 | N2 | t | DF |
|  |  |  |  |  |  |  |  |  |
| -RU486 - +RU486 |  |  |  |  |  |  |  |  |
| -Paraquat | 0.2723 | 0.2115 | 0.06075 | 0.1036 | 4 | 4 | 0.5867 | 12.00 |
| +Paraquat | 0.6493 | 0.4615 | 0.1878 | 0.1036 | 4 | 4 | 1.813 | 12.00 |

**Figure 3. Paraquat Assays 1+ 2 combined data. 2-way ANOVA, Rows are Paraquat, Columns are RU486**

| Table Analyzed | Grouped: Two-way ANOVA (two data sets) |  |  |  |  |
| --- | --- | --- | --- | --- | --- |
|  |  |  |  |  |  |
| Two-way ANOVA | Ordinary |  |  |  |  |
| Alpha | 0.05 |  |  |  |  |
|  |  |  |  |  |  |
| Source of Variation | % of total variation | P value | P value summary | Significant? |  |
| Interaction | 0.1352 | 0.7517 | ns | No |  |
| Row Factor | 59.33 | <0.0001 | **** | Yes |  |
| Column Factor | 3.468 | 0.1168 | ns | No |  |
|  |  |  |  |  |  |
| ANOVA table | SS | DF | MS | F (DFn, DFd) | P value |
| Interaction | 0.001861 | 1 | 0.001861 | F (1, 28) = 0.1021 | P=0.7517 |
| Row Factor | 0.8166 | 1 | 0.8166 | F (1, 28) = 44.81 | P<0.0001 |
| Column Factor | 0.04774 | 1 | 0.04774 | F (1, 28) = 2.620 | P=0.1168 |
| Residual | 0.5103 | 28 | 0.01822 |  |  |
|  |  |  |  |  |  |
| Difference between column means |  |  |  |  |  |
| Mean of -RU486 | 0.4609 |  |  |  |  |
| Mean of +RU486 | 0.3836 |  |  |  |  |
| Difference between means | 0.07725 |  |  |  |  |
| SE of difference | 0.04773 |  |  |  |  |
| 95% CI of difference | -0.02052 to 0.1750 |  |  |  |  |
|  |  |  |  |  |  |
| Difference between row means |  |  |  |  |  |
| Mean of -Paraquat | 0.2625 |  |  |  |  |
| Mean of +Paraquat | 0.5820 |  |  |  |  |
| Difference between means | -0.3195 |  |  |  |  |
| SE of difference | 0.04773 |  |  |  |  |
| 95% CI of difference | -0.4173 to -0.2217 |  |  |  |  |
|  |  |  |  |  |  |
| Interaction CI |  |  |  |  |  |
| Mean diff, A1 - B1 | 0.06200 |  |  |  |  |
| Mean diff, A2 - B2 | 0.09250 |  |  |  |  |
| (A1 -B1) - (A2 - B2) | -0.03050 |  |  |  |  |
| 95% CI of difference | -0.2260 to 0.1650 |  |  |  |  |
| (B1 - A1) - (B2 - A2) | 0.03050 |  |  |  |  |
| 95% CI of difference | -0.1650 to 0.2260 |  |  |  |  |
|  |  |  |  |  |  |
| Data summary |  |  |  |  |  |
| Number of columns (Column Factor) | 2 |  |  |  |  |
| Number of rows (Row Factor) | 2 |  |  |  |  |
| Number of values | 32 |  |  |  |  |

Pairwise comparisons with Bonferroni correction

| Compare each cell mean with the other cell mean in that row |  |  |  |  |  |  |  |  |
| --- | --- | --- | --- | --- | --- | --- | --- | --- |
|  |  |  |  |  |  |  |  |  |
| Number of families | 1 |  |  |  |  |  |  |  |
| Number of comparisons per family | 2 |  |  |  |  |  |  |  |
| Alpha | 0.05 |  |  |  |  |  |  |  |
|  |  |  |  |  |  |  |  |  |
| Bonferroni's multiple comparisons test | Mean Diff. | 95.00% CI of diff. | Below threshold? | Summary | Adjusted P Value |  |  |  |
|  |  |  |  |  |  |  |  |  |
| -RU486 - +RU486 |  |  |  |  |  |  |  |  |
| -Paraquat | 0.06200 | -0.09787 to 0.2219 | No | ns | 0.7324 |  |  |  |
| +Paraquat | 0.09250 | -0.06737 to 0.2524 | No | ns | 0.3629 |  |  |  |
|  |  |  |  |  |  |  |  |  |
|  |  |  |  |  |  |  |  |  |
| Test details | Mean 1 | Mean 2 | Mean Diff. | SE of diff. | N1 | N2 | t | DF |
|  |  |  |  |  |  |  |  |  |
| -RU486 - +RU486 |  |  |  |  |  |  |  |  |
| -Paraquat | 0.2935 | 0.2315 | 0.06200 | 0.06750 | 8 | 8 | 0.9185 | 28.00 |
| +Paraquat | 0.6283 | 0.5358 | 0.09250 | 0.06750 | 8 | 8 | 1.370 | 28.00 |
