# Supplementary material for: Mifepristone Increases Life Span of Virgin Female Drosophila on Regular and High-fat Diet Without Reducing Food Intake
Source: Front Genet. 2021 Sep 24;12:751647. doi: 10.3389/fgene.2021.751647 (PMC8511958; doi:10.3389/fgene.2021.751647)
Supplement: Supplementary file 7 [file DataSheet1.docx]

**Table S1. COX proportional hazards analyses**

RU = mifepristone/RU486

CO = coconut oil

**Figure 2 CO Assay 1**

Call: coxph(formula = (Surv(Date) ~ RU * CO), data = cox9402)

n= 591, number of events= 591

coef exp(coef) se(coef) z Pr(>|z|)

RU -0.87164 0.41827 0.13250 -6.578 4.76e-11 ***

CO 0.55766 1.74658 0.03422 16.297 < 2e-16 ***

RU:CO 0.08640 1.09024 0.03795 2.277 0.0228 *

Signif. codes: 0 ‘***’ 0.001 ‘**’ 0.01 ‘*’ 0.05 ‘.’ 0.1 ‘ ’ 1

exp(coef) exp(-coef) lower .95 upper .95

RU 0.4183 2.3908 0.3226 0.5423

CO 1.7466 0.5725 1.6333 1.8677

RU:CO 1.0902 0.9172 1.0121 1.1744

Concordance= 0.773 (se = 0.011 )

Likelihood ratio test= 500.4 on 3 df, p=<2e-16

Wald test = 410.2 on 3 df, p=<2e-16

Score (logrank) test = 527.9 on 3 df, p=<2e-16

**Figure 2 CO Assay 2**

Call: coxph(formula = (Surv(Date) ~ RU * CO), data = cox9403)

n= 559, number of events= 559

coef exp(coef) se(coef) z Pr(>|z|)

RU -1.03101 0.35665 0.14769 -6.981 2.94e-12 ***

CO 0.53916 1.71457 0.03507 15.375 < 2e-16 ***

RU:CO 0.11956 1.12701 0.04084 2.927 0.00342 **

Signif. codes: 0 ‘***’ 0.001 ‘**’ 0.01 ‘*’ 0.05 ‘.’ 0.1 ‘ ’ 1

exp(coef) exp(-coef) lower .95 upper .95

RU 0.3566 2.8039 0.267 0.4764

CO 1.7146 0.5832 1.601 1.8366

RU:CO 1.1270 0.8873 1.040 1.2209

Concordance= 0.764 (se = 0.012 )

Likelihood ratio test= 474.1 on 3 df, p=<2e-16

Wald test = 385.5 on 3 df, p=<2e-16

Score (logrank) test = 481.2 on 3 df, p=<2e-16

**Figure 2 CO Assays 1 and 2 combined**

Call: coxph(formula = (Surv(Date) ~ RU * CO), data = alldata)

n= 1150, number of events= 1150

coef exp(coef) se(coef) z Pr(>|z|)

RU -0.96648 0.38042 0.09887 -9.775 < 2e-16 ***

CO 0.54892 1.73138 0.02451 22.398 < 2e-16 ***

RU:CO 0.10679 1.11270 0.02779 3.842 0.000122 ***

Signif. codes: 0 ‘***’ 0.001 ‘**’ 0.01 ‘*’ 0.05 ‘.’ 0.1 ‘ ’ 1

exp(coef) exp(-coef) lower .95 upper .95

RU 0.3804 2.6287 0.3134 0.4618

CO 1.7314 0.5776 1.6502 1.8166

RU:CO 1.1127 0.8987 1.0537 1.1750

Concordance= 0.768 (se = 0.008 )

Likelihood ratio test= 980.5 on 3 df, p=<2e-16

Wald test = 798.9 on 3 df, p=<2e-16

Score (logrank) test = 1012 on 3 df, p=<2e-16

**Figure 3 Paraquat Assay 1**

Call: coxph(formula = (Surv(Day) ~ Paraquat * RU), data = cox9400)
  n= 374, number of events= 374
               coef exp(coef) se(coef)      z Pr(>|z|)    
Paraquat     2.4765   11.8997   0.2250 11.007  < 2e-16 ***
RU          -1.8165    0.1626   0.1989 -9.133  < 2e-16 ***
Paraquat:RU   1.4824    4.4036   0.2463  6.019 1.76e-09 ***
Signif. codes:  0 ‘***’ 0.001 ‘**’ 0.01 ‘*’ 0.05 ‘.’ 0.1 ‘ ’ 1
            exp(coef) exp(-coef) lower .95 upper .95
Paraquat       11.8997    0.08404    7.6563   18.4951
RU             0.1626    6.15040    0.1101    0.2401
Paraquat:RU    4.4036    0.22709    2.7175    7.1360
Concordance= 0.748  (se = 0.015 )
Likelihood ratio test= 379.9  on 3 df,   p=<2e-16
Wald test            = 239  on 3 df,   p=<2e-16
Score (logrank) test = 329.9  on 3 df,   p=<2e-16

**Figure 3 Paraquat Assay 2**

Call: coxph(formula = (Surv(Day) ~ Paraquat * RU), data = cox9401)
  n= 390, number of events= 390
                coef exp(coef) se(coef)      z Pr(>|z|)    
Paraquat     3.26041  26.06031  0.24761 13.168   <2e-16 ***
RU           0.05773   1.05943  0.14851  0.389    0.697    
Paraquat:RU -0.11519   0.89119  0.20622 -0.559    0.576    
Signif. codes:  0 ‘***’ 0.001 ‘**’ 0.01 ‘*’ 0.05 ‘.’ 0.1 ‘ ’ 1
            exp(coef) exp(-coef) lower .95 upper .95
Paraquat       26.0603    0.03837   16.0405    42.339
RU             1.0594    0.94390    0.7919     1.417
Paraquat:RU    0.8912    1.12209    0.5949     1.335
Concordance= 0.716  (se = 0.012 )
Likelihood ratio test= 336.1  on 3 df,   p=<2e-16
Wald test            = 197.9  on 3 df,   p=<2e-16
Score (logrank) test = 326.9  on 3 df,   p=<2e-16

**Figure 3 Paraquat Assays 1+2 combined**

Call: coxph(formula = (Surv(Day) ~ Paraquat * RU), data = alldata)
  n= 764, number of events= 764
               coef exp(coef) se(coef)      z Pr(>|z|)    
Paraquat     2.8644   17.5394   0.1658 17.277  < 2e-16 ***
RU          -0.6140    0.5412   0.1069 -5.746 9.16e-09 ***
Paraquat:RU   0.4147    1.5139   0.1469  2.822  0.00477 **
Signif. codes:  0 ‘***’ 0.001 ‘**’ 0.01 ‘*’ 0.05 ‘.’ 0.1 ‘ ’ 1
            exp(coef) exp(-coef) lower .95 upper .95
Paraquat       17.5394    0.05701   12.6732   24.2741
RU             0.5412    1.84776    0.4389    0.6673
Paraquat:RU    1.5139    0.66055    1.1351    2.0191
Concordance= 0.738  (se = 0.01 )
Likelihood ratio test= 658.5  on 3 df,   p=<2e-16
Wald test            = 413.4  on 3 df,   p=<2e-16
Score (logrank) test = 631  on 3 df,   p=<2e-6
